# Two transcription factors TaPpm1 and TaPpb1 co-regulate anthocyanin biosynthesis in purple pericarps of wheat

**DOI:** 10.1093/jxb/ery101

**Published:** 2018-03-17

**Authors:** Wenhui Jiang, Tianxiang Liu, Wenzhi Nan, Diddugodage Chamila Jeewani, Yanlu Niu, Chunlian Li, Yong Wang, Xue Shi, Cong Wang, Jiahuan Wang, Yang Li, Xin Gao, Zhonghua Wang

**Affiliations:** State Key Laboratory of Crop Stress Biology for Arid Areas, College of Agronomy, Northwest A&F University, Yangling, Shaanxi, China

**Keywords:** Allelic variants, anthocyanin synthesis, bHLH, R2R3-MYB, purple pericarp, tandem repeats in promoters, transcriptional regulation, wheat

## Abstract

Purple pericarps of bread wheat (*Triticum aestivum* L.) are a useful source of dietary anthocyanins. Previous mapping results indicated that the purple pericarp trait is controlled by two complementary genes located on chromosomes 7D and 2A. However, the identity of the genes and the mechanisms by which they regulate the trait are unknown. In this study, two transcription factors were characterised as anthocyanin activators in purple pericarps: TaPpm1 (purple pericarp-MYB 1) and TaPpb1 (purple pericarp-bHLH 1). Three non-functional variants were detected in the coding sequence of *TaPpm1* from non-purple seed lines, in which the function of *TaPpm1* was destroyed either by insertion-induced frame shifts or truncated peptides. There were six 261-bp tandem repeats in the promoter region of *TaPpb1* in the purple-grained varieties, while there was only one repeat unit present in the non-purple varieties. Furthermore, using yeast two-hybrid, dual luciferase, yeast one-hybrid, and transient assays, we were able to demonstrate that the interaction of TaPpm1 and TaPpb1 co-regulates the synthesis of anthocyanin. Overall, our results provide a better understanding of the molecular basis of anthocyanin synthesis in the wheat pericarp and indicate the existence of an integrated regulatory mechanism that controls production.

## Teaser

Two genes controlling the purple pericarp trait in wheat, *TaPpm1* and *TaPpb1*, are identified and the mechanism by which they co-regulate anthocyanin synthesis is proposed.

## Introduction

Anthocyanins are a class of plant flavonoid compounds that exist in the flowers, leaves, fruit, and seeds of many plant species. They have attracted widespread interest due to their various biological functions, including playing protective roles against environmental stress and providing beneficial effects on human health (Sarma and Sharma, 1999; Petroni and Tonelli, 2011). In many plants, the anthocyanin biosynthetic pathway has been well characterized and the corresponding genes have been isolated (Winkel-Shirley, 2001). This biosynthesis process is transcriptionally regulated by the MBW complex of R2R3-MYB, bHLH, and WD40 proteins (Koes *et al.*, 2005). WD40 acts as a docking platform involved in facilitating both the interaction of MYB and bHLH and the stabilization of the transcription factor (TF) complex, but it has no intrinsic enzymatic functions (DNA-binding or initiating target genes) (Ramsay and Glover, 2005; Hichri *et al.*, 2010). Plant MYBs are a large family with members participating in various physiological and metabolic processes (Stracke *et al.*, 2001). Among them, R2R3-MYB contains two imperfect repeats, R2 and R3, in the N-terminus, and R2R3-MYB is the most common type in regulating anthocyanin synthesis (Nesi *et al.*, 2001). Examples are *Rosea1* in snapdragon (Schwinn *et al.*, 2006), *AN1* in potato (D’Amelia *et al.*, 2014), and *PyMYB114* in pear (Yao *et al.*, 2017). The conserved R3 repeat is involved in interacting with bHLH co-factors, while the C-terminus that regulates the target gene expression is variable (Hichri *et al.*, 2011). Together with MYB, the bHLH proteins are crucial transcriptional regulators in the anthocyanin pathway; examples are TT8 in Arabidopsis (Nesi *et al.*, 2000), Delia in snapdragon (Martin *et al.*, 1991), and AN1 in petunia (Spelt *et al.*, 2000). The bHLH protein consists of a basic region for DNA-specific binding and a HLH region that allows homo- or heterodimer formation. In Arabidopsis, bHLH proteins activate the anthocyanin pathway and are grouped into the IIIf clade (Heim *et al.*, 2003). Moreover, these TFs are characterized by their specific temporal and spatial expression, and they are regulated by both the external environment and by plant development, resulting in distinct colour distribution in tissues (Ramsay and Glover, 2005; Jaakola, 2013).

In previous studies, mutations have often been detected in the promoters or coding sequences of the bHLH or MYB proteins, and these have effects on the ability of TFs to activate the downstream structural genes. These variations are ultimately responsible for diverse colour distributions or intensities (Butelli *et al.*, 2012; Rahman *et al.*, 2013; Cheng *et al.*, 2015). A *Copia*-like retrotransposon inserted in the promoter region of grape, *VvmybA1*, blocks gene expression and causes a colour deficiency in the skin of the berry (Kobayashi *et al.*, 2004). In sweet cherry, the formation of different coloured fruit (dark-red, blush, and yellow) is due to different combinations of the *PavMYB10.1* alleles *PavMYB10.1a* and mutated *PavMYB10.1b* (Jin *et al.*, 2016). In addition, a hierarchical and feedback regulation within the MBW complex also influences the expression pattern of TF genes and colour pigmentation (Baudry *et al.*, 2006; Li *et al.*, 2016). For example, a MYB–bHLH (JAF13 type) dimer in Solanaceae can regulate the transcript of the bHLH gene *AN1*, which subsequently participates in regulating the downstream structural genes in the anthocyanin pathway (Montefiori *et al.*, 2015). In monkeyflower, the self-activation of the MYB gene *NEGAN*, combined with *MlANbHLH1* and *MlWD40a*, is thought to be critical for generating anthocyanin spots (Yuan *et al.*, 2014).

In wheat (*Triticum aestivum* L.), anthocyanin appears in many areas, such as the coleoptile, leaf, glume, stem, anther, and pericarp. Various colours of wheat pericarp occur in different varieties, including white, red, blue, and purple. As anthocyanins have the ability to scavenge free radicals that cause oxidative stress in human cells (Bartłomiej *et al.*, 2012), purple-grained varieties enriched with bioactive anthocyanin compounds in the pericarps are considered as useful germplasm in breeding programmes (Hosseinian *et al.*, 2008). In addition, purple colouration is also treated as a visible morphological marker during the selection of other important economical traits (Bagge *et al.*, 2007; Khlestkina, 2013). In previous studies, the relevant anthocyanin regulatory genes have been considered to be MYB and bHLH TFs (Li *et al.*, 1999; Khlestkina, 2013; Shin *et al.*, 2016). Two loci, *Pp-D1* on chromosome 7D and *Pp3* on chromosome 2A were identified as regulating anthocyanin biosynthesis in purple pericarps (Tereshchenko *et al.*, 2012). Based on the mapping information, a purple pericarp-related bHLH gene *Tamyc1* (KJ747954) was isolated, which was highly expressed in purple pericarps (Shoeva *et al.*, 2014). However, the exact genetic basis governing purple pericarps remains to be elucidated and few studies have been carried out to clarify the molecular mechanisms of anthocyanin formation in the pericarp of wheat.

Here, two transcription factor genes, *TaPpm1* and *TaPpb1*, were screened out from the wheat variety ‘Heixiaomai 76’ (H76) based on RNA-sequencing, expression analysis, and genetic mapping. Variants of these two genes were characterised in different varieties and specific markers were developed to genotype the different pericarp colour lines. The regulatory mechanisms underlying anthocyanin synthesis in the wheat pericarp were thus revealed.

## Materials and methods

### Plant material and extraction of RNA and DNA

Heixiaomai 76 (H76), a purple-grained variety of wheat (*Triticum aestivum* L.), was used for transcriptome sequencing. A white-grained variety, A14, was used for quantitative analysis of anthocyanin content and gene expression analysis, thus providing a contrast with H76. To identify the variations of *TaPpm1* and *TaPpb1*, corresponding fragments were amplified and sequenced from 12 wheat accession lines (two purple-, two blue-, and eight white-grained lines). In addition, 22 wheat lines (two purple- and 20 white-grained lines) were used to detect the distribution of sequence variations of *TaPpm1* and *TaPpb1*. A genetic mapping population including a total of 126 F_2_ progenies was constructed from a cross of A14 and H76. Seeds were planted in the field at the Northwest A&F University, Shaanxi, China. DNA was extracted using the CTAB method. RNA was isolated from 50-mg samples with an RNA isolation kit (TIANGEN, Beijing, China). cDNA was obtained by a reverse-transcription reaction using the PrimeScript^TM^ RT kit (TaKaRa Biotech, Dalian, China).

### Transcriptome sequencing and functional annotation

Transcriptome analyses were conducted on H76 pericarps collected at two developmental stages under different conditions. A sample of grains with the glumes removed at 10 days after pollination (DAP) were directly exposed to sunlight (designated as the sample under high-light treatment). Grains without any treatment were designated as samples under normal conditions. After exposure to sunlight for 12 h, the pericarps were peeled from the grains without glumes and labelled as H-10p. Pericarps were also peeled from grains at 10 DAP and 17 DAP under normal conditions and labelled as H-10d and H-17d, respectively. Libraries were constructed and sequenced using an Illumina HiSeq™ 2000 system (San Diego, CA, USA). Raw reads were cleaned by removing the adaptor sequences and low-quality sequences, and were then used for *de novo* transcriptome assembly using the Trinity software (https://github.com/trinityrnaseq/trinityrnaseq/wiki). Functional annotation of the unigenes was performed using the NCBI non-redundant (nr) database (http://www.ncbi.nlm.nih.gov), the Swiss-Prot protein database (http://www.expasy.ch/sprot), and the Kyoto Encyclopedia of Genes and Genomes database (Kanehisa *et al.*, 2004). Blast2GO was employed to obtain the relevant GO terms based on the nr BLAST results (Conesa *et al.*, 2005).

### Analysis of differentially expressed genes

Transcript levels were calculated using the RPKM method (reads per kb per million reads). Genes with *P*≤0.01, RPKM≥2, and an absolute value of the log_2_ ratio≥1 were marked as differentially expressed genes (DEGs). The anthocyanin-related genes selected from the DEGs were mapped to several databases: the wheat genome and cDNA database (Triticum_aestivum.TGACv1, http://plants.ensembl.org/index.html) and the wheat genome donor database (http://wheat-urgi.versailles.inra.fr/Seq-Repository). The scaffold that aligned best with the chromosome information was used to determine the location of the unigenes.

### Phylogenetic analysis

The unigenes were translated using the gene prediction software FGENESH (Solovyev *et al.*, 2006). The conserved domains of the bHLHs were predicted using the SMART program (Schultz *et al.*, 2000). Sequences of Arabidopsis R2R3-MYB proteins were retrieved from the Arabidopsis Genome Annotation version 7.0 (http://www.arabidopsis.org). In addition, AtbHLH sequences were retrieved from the UniProt Database (http://www.uniprot.org). The amino acids of the MYB proteins and the conserved domains of bHLH proteins were used to perform phylogenetic analysis using MEGA (version 5.02) (Tamura *et al.*, 2011) with the neighbour-joining statistical method and 1000 bootstrap replicates.

### Quantitative analysis of anthocyanin content

The content of total anthocyanins was measured using the colorimetric method with different pH solutions (Ficco *et al.*, 2014). Two dilutions of the samples (0.1 g) were prepared, one at pH 1.0 using potassium chloride buffer (0.03 M KCl) and the other using sodium acetate buffer (0.4 M CH_3_CO_2_Na.3H_2_O) at pH 4.5. The absorbance of each sample was measured at 520 nm and 710 nm against distilled water as a blank. The total anthocyanin content was corrected for dry matter and expressed as Cyanidin-3-Glucoside equivalents in μg g^–1^ dry matter.

### Expression analysis of regulatory genes and anthocyanin-related structural genes

Five types of tissues of H76 and A14 were collected: pericarps at 10, 16, 22, and 28 DAP, coleoptiles at 3 d after germination, second topmost leaves at the heading stage, and auricles and glumes at the grain-filling stage. For the F_2_ population from the cross between A14 and H76, pericarps were collected at 20 DAP. Transcription of regulatory genes (*TaPpm1*, *TaPpm2*, *TaPpm3*, and *TaPpb1*) and structural genes, selected from transcriptome data, were quantified by quantitative real-time PCR (qRT-PCR) using THUNDERBIRD qPCR Mix (TOYOBO, Japan) and an ABI 7500 Real-Time PCR System (Life Technologies). Primers are listed in Supplementary Table S1 at *JXB* online. qRT-PCR was conducted by denaturing at 95 °C for 1 min, followed by 40 cycles of 95 °C for 5 s, 60 °C for 15 s, and 72 °C for 30 s. The data were analysed with the 2^−△△*C*T^ method (Livak and Scmittgen, 2001), and the qRT-PCR reactions were normalized with *TaActin* in wheat. All biological replicates were measured in triplicate.

### Gene cloning and sequence analysis

The promoter and genomic fragments of *TaPpm1* and *TaPpb1* in the 12 selected varieties (two purple-, two blue-, and eight white-grained lines) were amplified and sequenced. cDNAs designated *TaPpm1a*, *TaPpm1b*, *TaPpm1c*, and *TaPpm1d* were amplified from the varieties H76, Hedongwumai, Bobwhite, and A14, respectively. The cDNA of *TaPpm2* and *TaPpm3* were amplified from H76. The cDNA of *TaPpb1a* and *TaPpb1b* were amplified from H76 and A14, respectively. The promoters of *TaPpb1a* and *TaPpb1b* were amplified from H76 and A14, respectively. The PCR mixture (20 μl) contained 1 μl of genomic DNA or cDNA, 2× KOD buffer, 2 mM dNTP, 0.5 μM primers, 3 μl ddH_2_O, and 0.4 μl of KOD (TOYOBO, Japan). A total of 35 PCR cycles were performed, with each cycle involving 20 s at 98 °C, 15 s at 56–59 °C (according to the different *T*_m_ of primers), and 1–3.5 min at 68 °C. All primers are listed in Supplementary Table S1. Multiple sequence alignments were performed using ClustalX 1.83 (http://www.ebi.ac.uk). *Cis*-acting elements were predicted using the PlantCARE program (http://bioinformatics.psb.ugent.be/webtools/plantcare/html/).

### Chromosomal mapping of *TaPpm1* and *TaPpb1*

A total of 126 F_2_ plants from the cross between A14 and H76 were used for chromosomal mapping. Thirty SSR markers located on chromosome 7D and 28 SSR markers located on chromosome 2A were selected from GrainGenes (http://wheat.pw.usda.gov/GG3/) (Supplementary Table S1). The specific marker 7D02 and two sets of primers (2APRO1 and 2APRO2) were designed to distinguish the genotypes of *TaPpm1* and *TaPpb1*, respectively. The intermediate map ‘Wheat, Consensus SSR, 2004’ (Somers *et al.*, 2004) was employed to connect our genetic map of chromosome 2A with the corresponding map of the near-isogenic line ‘i:S29Pp1Pp3P’ ( ‘Saratovskaya 29’ with 7D and 2A introgression derived from the purple pericarp donor ‘Purple’) (Tereshchenko *et al.*, 2012). All linkage maps were constructed using the QTL IciMapping software (http://www.isbreeding.net/software/) and Mapdraw software (Liu and Meng, 2003).

### Subcellular localization analysis

*TaPpm1a* and *TaPpb1* were cloned into the pA7-GFP vector at the *Spe*I restriction site under the control of the CaMV35S promoter (Supplementary Table S1). Both fusion constructs (TaPpm1-GFP and TaPpb1-GFP) and empty vectors were transformed into living onion epidermal cells by particle bombardment. After incubation on Murashige and Skoog (MS) medium (pH 5.8) solidified with 3% agar at 28 °C for 24–48 h, the onion cells were observed using bright field and fluorescence confocal microscopy (Olympus, Tokyo, Japan).

### Yeast two-hybrid and one-hybrid assays

The coding sequences (CDSs) of *TaPpm1*s were cloned into pGADT7 using the *Eco*RI site. The coding regions of *TaPpb1* with different 3′-deletions (*TaPpb1*_*1707*_, *TaPpb1*_*1364*_, *TaPpb1*_*1065*_, and *TaPpb1*_*642*_) were cloned into pGBKT7 using the *Eco*RI site to detect their autoactivation. Yeast two-hybrid (Y2H) screening was performed according to the instructions for the Matchmaker^TM^ Gold Yeast Two-Hybrid System (Clontech). The PGADT7-TaPpm1 vectors and PGBKT7-TaPpb1 vectors were transfected into the yeast strains Y187 and Y2HGold, respectively. AD-TaPpm1s were mated with BD-TaPpb1_642_ and then cultured on a DDO (SD/–Leu/–Trp) plate for 4 d. Yeast cells transformed with AD-T and BD-Lam were used as negative controls, while yeast cells transformed with AD-T and BD-53 served as positive controls. Interaction screening was carried out on QDO (SD/–Leu/–Trp/–His/–Ade) plates and QDO/X/A (SD/–Leu/–Trp/–His/–Ade/X-α-Gal/AbA) plates.

For the yeast one-hybrid (Y1H) assay, a 254-bp promoter sequence (from –682 bp to –935 bp) of *TaPpm1* was cloned into a pAbAi Y1H bait vector, yielding TaPpm1pro-pAbAi. The different pairwise combinations of AD-TaPpm1s, BD-TaPpb1_642_, and corresponding empty vector controls were co-transformed into the Y1HGold strain containing the TaPpm1pro-pAbAi vector to detect their promoter binding ability. Growth on –Leu/–Trp/–Ura plates supplemented with 500 ng ml^–1^ aureobasidin A was scored after 3 d at 30 °C. Y1H screening was performed according to the instructions for the Yeast One-Hybrid System (Clontech).

### Transient assay in wheat callus

*TaPpm1*s (*TaPpm1a*, *TaPpm1b*, *TaPpm1c*, and *TaPpm1d*) and *TaPpb1* were inserted into the pCXSN vector (Chen *et al.*, 2009), driven by the CaMV35S promoter (Supplementary Table S1). Mature embryos of Chinese Spring were soaked in MS medium (containing 2 mg l^–1^ 2,4-D and 30 g l^–1^ sucrose) and cultured for 2 d in the dark to produce calluses. Vectors were introduced into these calluses by particle bombardment. After 1 d in the dark followed by 1 d in the light, purple dots could be observed the next day. Calluses were collected and used for qRT-PCR to detect the regulatory and anthocyanin-related structural genes. The qRT-PCR procedure was the same as that mentioned above.

### Dual luciferase assay

The promoter regions of *TaPpb1a*, *TaPpb1b*, and *ANS* (anthocyanidin synthase) were cloned into pGreenII 0800-LUC, yielding luciferase reporter constructs. Genes encoding *TaPpm1a*, *TaPpm1b*, and *TaPpb1* were cloned into the pGreenII 62SK binary vectors (Supplementary Table S1). All constructs were transformed into *Agrobacterium tumefaciens* GV3101. Leaves of *Nicotiana benthamiana* (4 weeks old) were infiltrated with mixed agrobacteria and collected for transient transcription assays according to the method described previously by Hellens *et al.* (2005).

## Results

### Identification of differentially expressed genes involved in the anthocyanin pathway

**Fig. 1.**
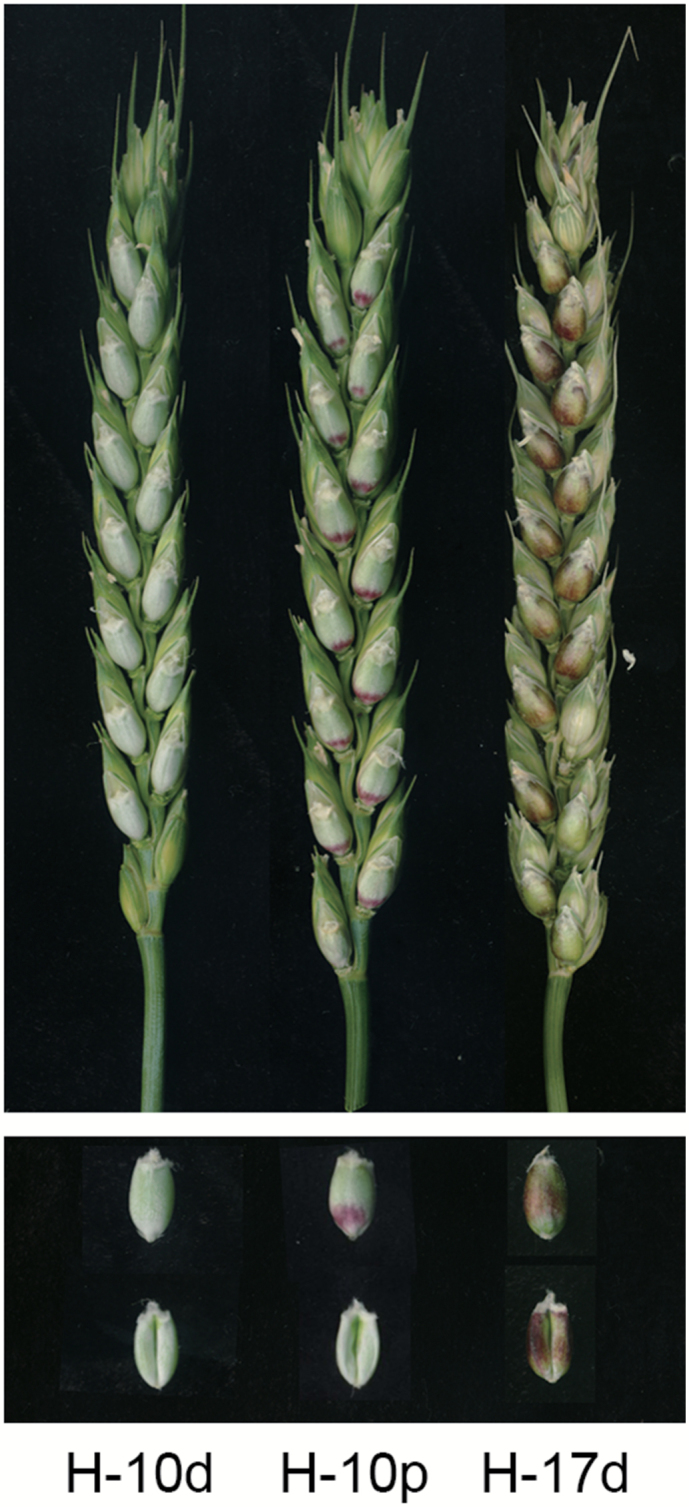
Pericarp samples of variety H76 at different stages under normal and high-light conditions. H-10d, 10 d after pollination (DAP) under normal conditions; H-10p, 10 DAP directly exposed in sunlight for 12 h; H-17d, 17 DAP under normal conditions. Both H-10p and H-17d had anthocyanin accumulation in the pericarps.

Anthocyanins were abundant in the pericarp of H76, and they increased with seed development and in response to high light (Fig. 1). Two complementary genes were indicated as controlling the purple pericarp of H76 (Supplementary Table S2). To identify these genes, high-throughput RNA-sequencing was performed to obtain a broad view of DEGs between the purple pericarp samples (H-10p and H-17d) and a non-purple pericarp sample (H-10d) (Fig. 1).

Gene expression was measured in non-purple and purple pericarps (H-10d vs. H-10p and H-10d vs. H-17d) (Supplementary Table S3) and as a result, 1833 DEGs were identified (Supplementary Table S4). Anthocyanin-related structural and regulatory genes (MYB and bHLH) were screened out from the DEGs. A total of 105 unigenes distributed among 18 kinds of structural genes were distinguished from the upregulated DEGs (Supplementary Table S5).

**Fig. 2.**
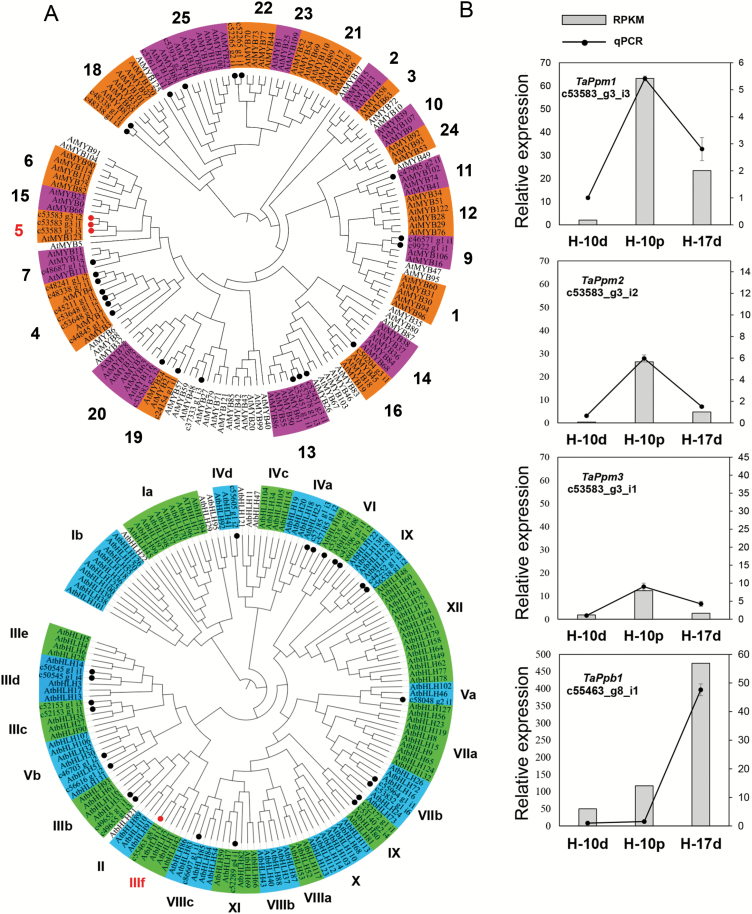
Phylogenetic analyses of the transcription factors (TFs) MYB and bHLH, and validation of the expression levels of four TFs related to anthocyanin synthesis in wheat. (A) A total of 26 TaMYBs (top) and 24 TabHLHs (bottom) protein sequences derived from wheat transcriptome data are labelled with dots. Proteins labelled with red dots belong to the clades of anthocyanin synthesis. (B) qRT-PCR validation of the four unigenes *c53583_g3_i3*, *c53583_g3_i2*, *c53583_g3_i1*, and *c53583_g8_i1* (designated as *TaPpm1*, *TaPpm2*, *TaPpm3*, and *TaPpb1*, respectively). Expression is relative to that of *TaActin*.

Phylogenetic analysis was employed to screen out the anthocyanin-related R2R3-MYB and bHLH proteins in the DEGs. A total of 125 Arabidopsis MYB TFs together with 26 wheat MYBs from the DEGs were divided into 23 subgroups (Stracke *et al.*, 2001) (Fig. 2A). Three unigenes (*c53583_g3_i3*, *c53583_g3_i2*, and *c53583_g3_i1*), designated *TaPpm1* (purple pericarp-MYB 1), *TaPpm2* (purple pericarp-MYB 2, GenBank accession No. MG066457), and *TaPpm3* (purple pericarp-MYB 3, GenBank accession No. MG066458), respectively, were identified from subgroup 5, where anthocyanin regulators *ZmC1* (maize) and *OsC1* (rice) are located (Schwinn *et al.*, 2016; Dubos *et al.*, 2010). One bHLH unigene (*c55463_g8_i1*), designated as *TaPpb1* (purple pericarp-bHLH 1), was clustered with AtMYB42 (AtTT8), an anthocyanin activator in Arabidopsis, in clade IIIf (Nesi *et al.*, 2000; Heim *et al.*, 2003) (Fig. 2A). These four genes were selected as candidate genes related to the purple pericarp trait. The expression profiles of these genes detected by RNA-sequencing were confirmed by qRT-PCR (Fig. 2B). This revealed that the expression levels of the three MYB genes were higher in the H-10p samples than in the H-17d samples. Among them, *TaPpm1* showed the highest expression. In contrast, *TaPpb1* exhibited a different expression profile, showing significantly higher expression in the H-17d samples compared with that in the H-10p samples.

In addition, the transcriptome sequences of *TaPpm1*, *TaPpm2*, *TaPpm3*, and *TaPpb1*, which were subjected to BLASTN searches in the wheat database, were mapped on the short arm of chromosome 7D, and the long arm of chromosomes 4B, 4D, and 2A, respectively (Supplementary Table S6). The chromosome locations of *TaPpm1* and *TaPpb1* were identical to those of loci identified as controlling purple pericarp in previous studies (Tereshchenko *et al.*, 2012; Khlestkina, 2013), thus indicating that they are candidate genes for the purple pericarp trait.

### Gene expression in different tissues and at different stages of seed development

**Fig. 3.**
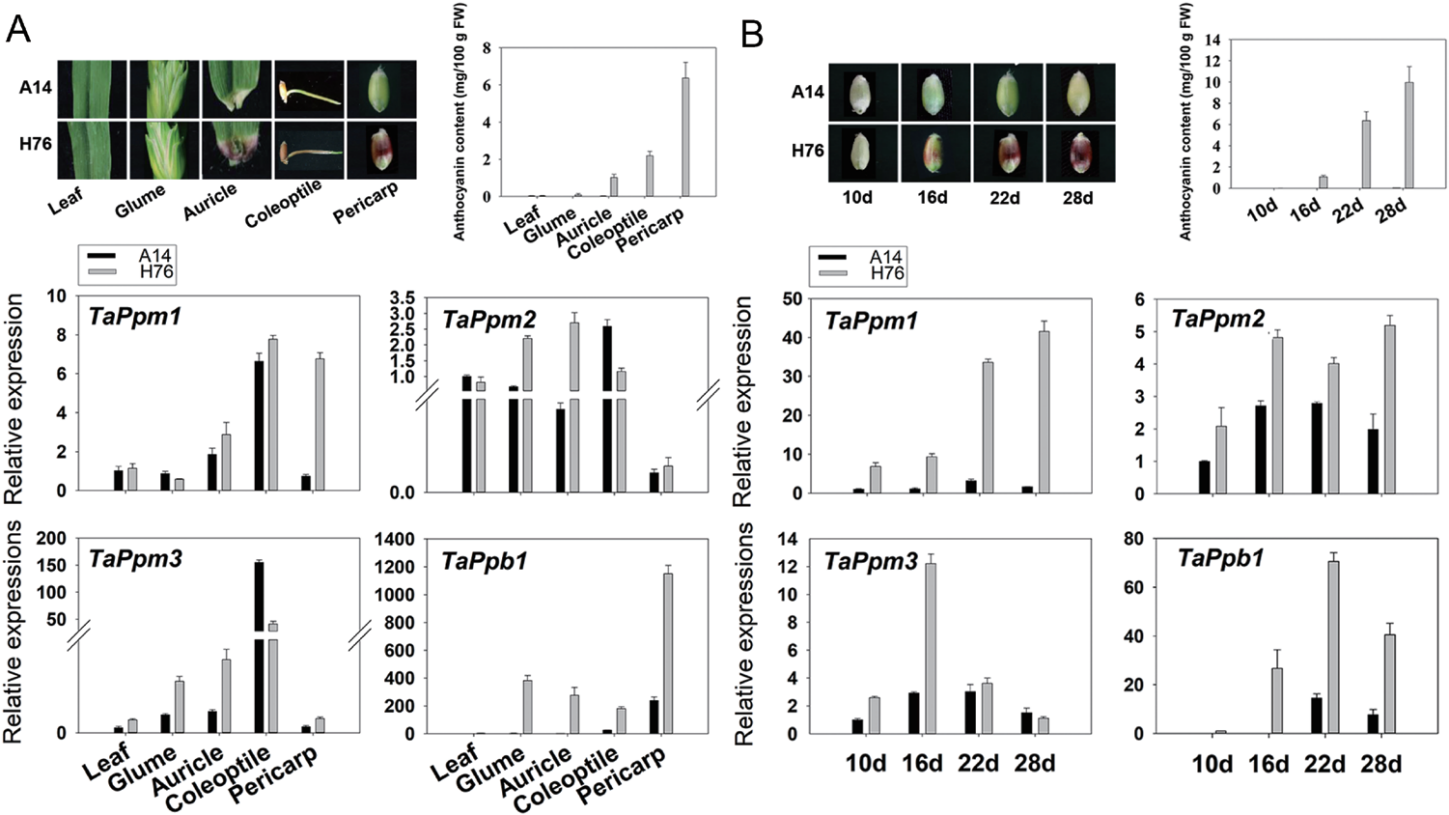
Expression patterns of the three wheat *TaPpm*s and *TaPpb1*. (A) Relative expression of transcription factor (TF) genes in different tissues of the two wheat lines, A14 (white pericarp) and H76 (purple pericarp). (B) Relative expression of TF genes at four physiological stages (days after pollination) during pericarp development. Data are means (±SD) of three biological replicates. Expression is relative to that of *TaActin*.

qRT-PCR was conducted to investigate the relationship between four genes (*TaPpm1*, *TaPpm2*, *TaPpm3*, and *TaPpb1*) and pericarp colour in five tissues of A14 and H76 (leaf, auricle, coleoptile, pericarp, and sheath) and at four seed development stages (10, 16, 22, and 28 DAP). None of the tissues of A14 contained anthocyanin, and neither did the leaves of H76. The pericarp of H76 exhibited the highest content of anthocyanin, with a tendency for it to increase during grain development (Fig. 3). Most of the detected structural genes had significantly higher expression in the purple pericarp than in other non-purple tissues (Supplementary Fig. S1A).

*TaPpm1* and *TaPpb1* were highly expressed in purple pericarps, whereas *TaPpm2* and *TaPpm3* showed lower expression in pericarps compared with levels in other tissues (Fig. 3A). At four stages of pericarp development, the expression profile of *TaPpm1* was consistent with that of some structural genes (e.g. *CHS*, *F3H*, *F3’H* and *DFR*) (Fig. 3B; Supplementary Fig. S1B), reflecting the accumulation pattern of anthocyanin. The expression patterns of the other TF genes were different from the colour accumulation tendency in the pericarps. However, *TaPpb1* had significantly higher expression in the purple pericarps than in the non-purple ones at all stages, indicating that *TaPpb1* was also related to pigment accumulation in the pericarp.

Based on the expression profiles of these genes and their chromosome locations, it could be inferred that *TaPpm1* and *TaPpb1* were candidate genes for purple pericarps. Thus, these two genes were chosen for characterization in our subsequent experiments.

### Analysis of sequence variations of *TaPpm1* and *TaPpb1* in different-coloured wheat lines

The coding regions and promoter sequences of *TaPpm1* and *TaPpb1* were analysed in 12 different-coloured varieties (Supplementary Fig. S2). The results indicated that sequence variations existed in the coding region of *TaPpm1* and the promoter region of *TaPpb1*.

**Fig. 4.**
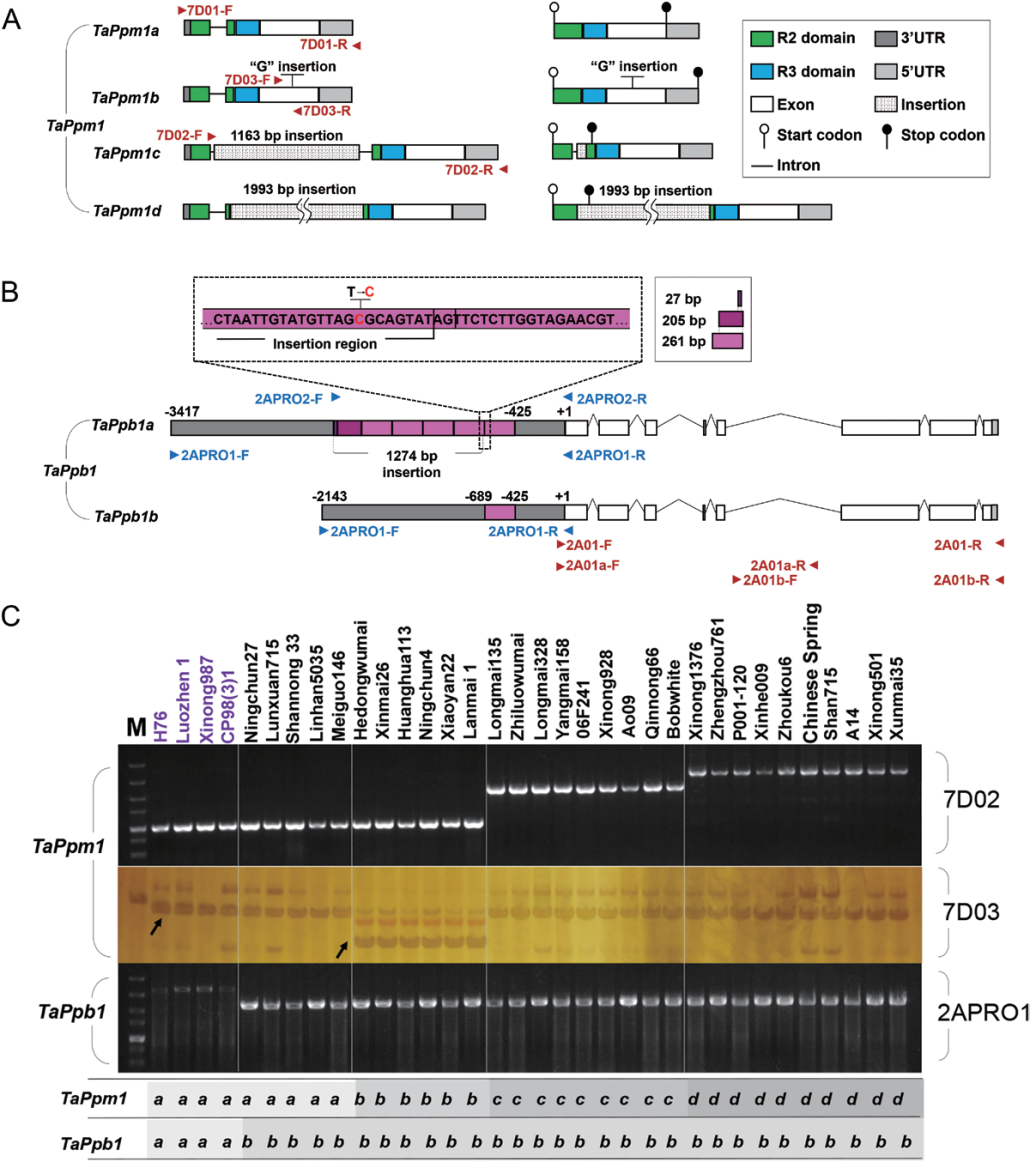
The variants of *TaPpm1* and *TaPpb1* and their distribution in the 34 wheat lines examined. (A) The genetic and transcript structures of the four variants of *TaPpm1*. (B) The genetic structures and the rearranged promoters of two *TaPpb1* variants (*TaPpb1a* and *TaPpb1b*). The purple boxes indicate the duplicated 261-bp units in the promoter region of *TaPpb1a* or *TaPpb1b*. In the fourth 261-bp unit, the 259-bp section belongs to the insertion region. The ‘C’ base, highlighted in red, in the fourth 261-bp unit is a transition from the ‘T’ base compared with other identical 261-bp units. (C) Different allelic variants of *TaPpm1* and *TaPpb1* were distinguished in 34 wheat varieties using specific markers. Varieties highlighted in purple have purple pericarps. The specific primer set 7D02 could distinguish *TaPpm1c* and *TaPpm1d* from the other two allelic variants (*TaPpm1a* and *TaPpm1b*). Amplicons of the dCAPS primer set 7D03 were digested by the *Dra*III enzyme to recognize *TaPpm1a* and *TaPpm1b* genotypes. *TaPpb1a* and *TaPpb1b* variants could be distinguished by 2APRO1.

In the coding region of *TaPpm1*, four variants were discovered and sequenced (Fig. 4A). In two purple varieties (H76 and Luozhen 1), the gene had an identical 872-bp gDNA sequence and a 759-bp full-length CDS, named *TaPpm1a* (GenBank accession No. MG066451). The second variant contained a 1-bp insertion at the 635 bp site in the C-terminus (*TaPpm1b*, GenBank accession No. MG066452) and its CDS was extended to 945 bp (Fig. 4A; Supplementary Fig. S3A). The third variant (*TaPpm1c*, GenBank accession No. MG066453) had a 1163-bp insertion, flanked by a 9-bp duplicated target site in the intron at the 156 bp site (Fig. 4A; Supplementary Fig. S3B). The CDS of *TaPpm1c* contained a 22-bp partial intron and a 64-bp insertion sequence, resulting in premature termination at the 261 bp site. The fourth variant (*TaPpm1d*, GenBank accession No. MG066454) had a 1993-bp insertion at the 272 bp site in the second exon. This insertion was a typical *Copia*-like retrotransposon containing an 855-bp ORF encoding a functional Gag-Pol polyprotein flanked by a 535-bp 5′ long terminal repeat (LTR) and 3′ LTR (Fig. 4A; Supplementary Fig. S3C). In the cDNA of *TaPpm1d*, the 1993-bp insertion fragment was maintained in the transcript after cutting off the intron, generating a 2752-bp transcript that prematurely terminated at the 273 bp site. Sequence alignment of TaPpm1 variants with several anthocyanin-related MYB proteins showed that some key motifs, such as the ‘bHLH interacting motif’ or ‘motif 5’ (related to anthocyanin biosynthesis) were lost in TaPpm1b, TaPpm1c, and TaPpm1d because of the sequence mutations (Supplementary Fig. S4A).

The coding region of *TaPpb1*, encoding an anthocyanin bHLH regulatory protein (Supplementary Fig. S4B), was identical in the 12 varieties. Mutations were only detected in its promoter, generating two variants of *TaPpb1* (*TaPpb1a/b*). *TaPpb1a* (GenBank accession No. MG066455), which was isolated from two purple varieties, had six 261-bp tandem repeat units in its promoter regions, while *TaPpb1b* (GenBank accession No. MG066456) had only one 261-bp unit (Fig. 4B; Supplementary Fig. S3D). The number of repeat units was inferred from the size of the PCR fragment (Supplementary Fig. S5).

Subsequently, 34 different-coloured lines were analysed for the genotypes of *TaPpm1* and *TaPpb1*, using specific markers that we developed. The *TaPpm1a* and *TaPpb1a* genotypes were detected in all the purple varieties (Fig. 4C), while *TaPpm1b*, *TaPpm1c*, *TaPpm1d*, and *TaPpb1b* existed only in the non-purple ones. These results suggested that *TaPpm1* and *TaPpb1* are closely associated with the phenotype of the purple pericarp in wheat.

### The association of the *TaPpm1* and *TaPpb1* genotypes with pericarp colours in hexaploid wheat

In this study, 126 F_2_ progenies from a cross between A14 (*TaPpm1d*/*TaPpm1d* and *TaPpb1b*/*TaPpb1b*) and H76 (*TaPpm1a*/*TaPpm1a* and *TaPpb1a*/*TaPpb1a*) were constructed in order to explore the relationship between pericarp phenotypes and the *TaPpm1* and *TaPpb1* genotypes. The F_1_ seeds displayed purple pericarp traits, reflecting the dominant nature of the purple phenotype. Among the 126 F_2_ individuals, 68 lines showed purple-coloured seeds and 58 lines showed white-coloured seeds, with the segregation data fitting with the ratio of 9:7 (purple:white) (χ^2^=0.31) (Supplementary Table S2). This indicated that pericarp colour was determined by two complementary genes, which was consistent with results obtained in previous studies (Tereshchenko *et al.*, 2012).Chromosomes 7D and 2A in our ‘A14×H76’ map were then compared with the corresponding chromosomes in the map of the near-isogenic line ‘i:S29Pp1Pp3P’ in order to verify the relationship between the candidate genes (*TaPpm1* and *TaPpb1*) and the previously identified loci (*Pp-D1* and *Pp3*). *TaPpm1* was mapped between *Xgwm44* and *Xgwm111*, where *Pp-D1* has been located (Supplementary Fig. S6A). *TaPpb1* was located between *Xwmc632* and *Xwmc47*, where *Pp3* has been located (flanked by *Xgwm328* and *Xgwm445*) (Tereshchenko *et al.*, 2012) (Supplementary Fig. S6B).

**Fig. 5.**
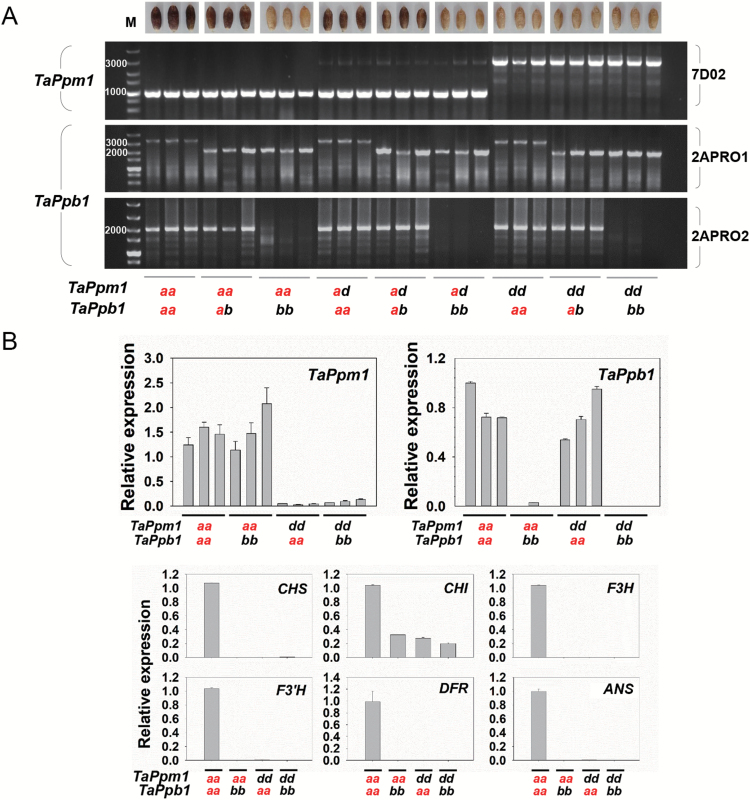
Association analysis between genotypes of *TaPpm1* and *TaPpb1* and the pericarp colours in F_2_ individuals. *TaPpm1a* and *TaPpb1a* highlighted in red are dominant to *TaPpm1d* and *TaPpb1b*, respectively. (A) Genotypes of *TaPpm1* and *TaPpb1* in different-coloured F_2_ progenies of the ‘A14×H76’ population. *TaPpm1a* presents a 967-bp sequence while *TaPpm1d* presents a 2960-bp fragment amplified with the specific primer set 7D02. *TaPpb1a* yields a 3417-bp fragment and *TaPpb1b* presents only a short 2143-bp fragment using 2APRO1. A 2143-bp and a 1996-bp fragment were amplified from heterozygous *TaPpb1* (*TaPpb1a*/*TaPpb1b*) by the primer sets 2APRO1 and 2APRO2, respectively. (B) The expression of *TaPpm1*, *TaPpb1*, and six structural genes was detected in F_2_ individuals with diverse genotypes of *TaPpm1* and *TaPpb1*. Expression is relative to that of *TaActin*.

Genotypes of *TaPpm1* and *TaPpb1* were associated with pericarp colours in the F_2_ progenies (Fig. 5A). The genotypes of the 58 white-pericarp progenies were at least one of either homozygous *TaPpm1d* or *TaPpb1b*. The genotypes of the remaining 68 purple-pericarp plants possessed both *TaPpm1a* and *TaPpb1a*. qRT-PCR analysis showed that *TaPpm1* and *TaPpb1* were highly expressed in individuals with dominant *TaPpm1a* or *TaPpb1a*, respectively, whereas six downstream structure genes were highly expressed only in lines carrying both *TaPpm1a* and *TaPpb1a* (Fig. 5B). Taken together, these results indicated that *TaPpm1* and *TaPpb1* are complementary genes that control downstream genes and subsequent purple pigment synthesis in wheat grains.

### Interactions between TaPpm1s and TaPpb1 proteins

**Fig. 6.**
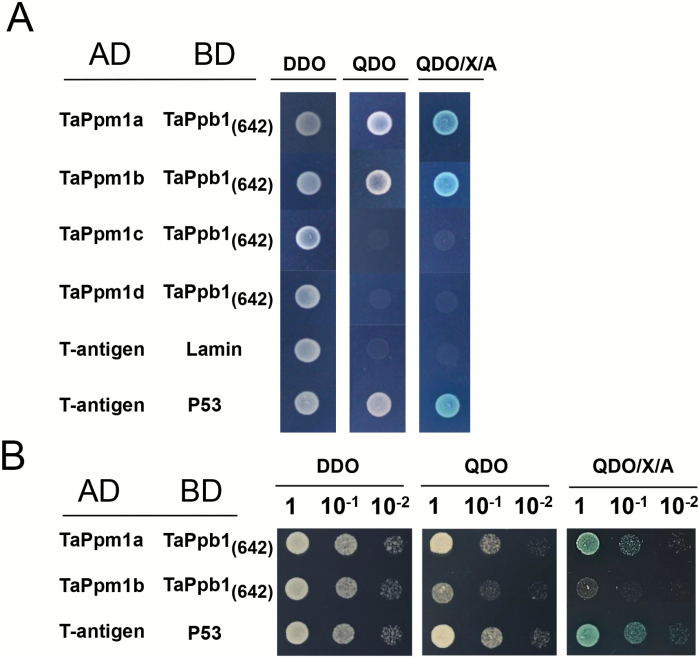
Detection of the interactions between TaPpm1s and TaPpb1 via yeast two-hybrid screening. (A) Interaction of TaPpm1s (TaPpm1a, TaPpm1b, TaPpm1c, and TaPpm1d) with truncated TaPpb1 protein in the yeast cells. *TaPpm1*s were fused to the GAL4 activation domain whereas a truncated form of *TaPpb1* (1–642 bp, encoding 214 amino acids) was fused to the GAL4 binding domain. Yeast clones transformed with different constructs were grown on DDO (SD/–Leu/–Trp), QDO (SD/–Leu/–Trp/–Ade/–His) and QDO/X/A (QDO supplemented with ABA and X-α-Gal) plates for 5 d. Yeast cells transformed with the AD-T-antigen and BD-Lamin were used as a negative control, while yeast cells transformed with the AD-T-antigen and BD-P53 served as a positive control. (B) Serial dilutions (10^–1^, 10^–2^) of the yeast cells with different combinations (AD-TaPpm1a with BD-TaPpb1_(642)_ or AD-TaPpm1b with BD-TaPpb1_(642)_) were cultured on DDO, QDO, or QDO/X/A for 1 d to detect their interaction abilities. (This figure is available in colour at *JXB* online.)

The subcellular localization assay showed TaPpm1 and TaPpb1 to be nuclear-localized proteins (Supplementary Fig. S7). The interaction between TaPpm1 and TaPpb1 was tested by Y2H assays. Y2HGold cells containing BD-TaPpb1_642_, which did not show any transcriptional activity in yeast cells, were selected to mate with Y187 harbouring AD-TaPpm1 (Supplementary Fig. S8). The results showed that TaPpm1a had a strong interaction with TaPpb1 (Fig. 6) while TaPpm1b had only a weak interaction, as yeast cells transformed with AD-TaPpm1b×BD-TaPpb1_642_ exhibited a slower plaque growth rate and a lighter X-α-gal colouration compared with those transformed with AD-TaPpm1a×BD-TaPpb1_642_ (Fig. 6B). TaPpm1c and TaPpm1d did not show interactions with TaPpb1 (Fig. 6A).

### Transient expression of *TaPpm1* and *TaPpb1* in wheat calluses

**Fig. 7.**
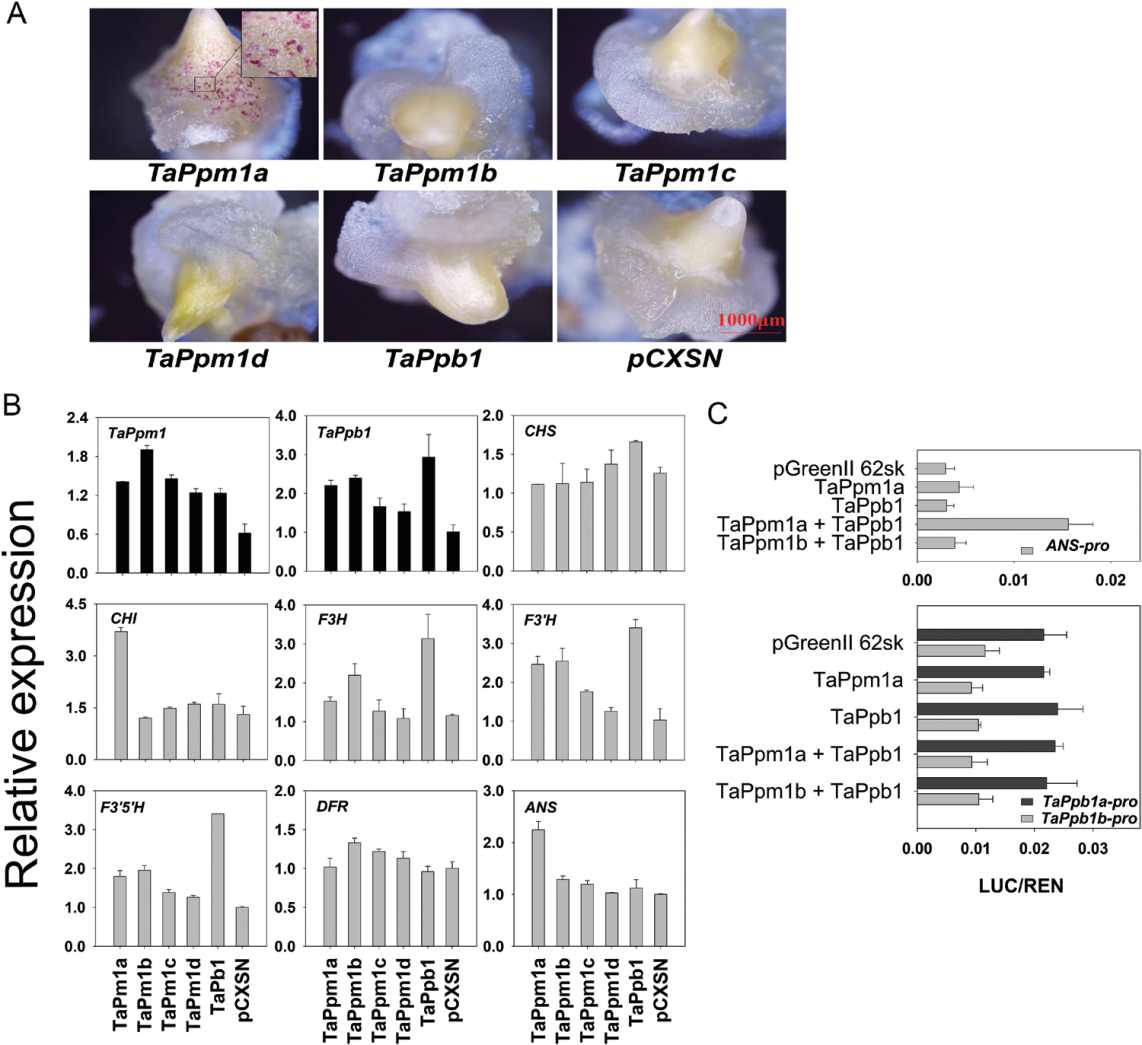
Transient expression of *TaPpm1*s and *TaPpb1* genes in wheat calluses and detection of their activation features by dual-luciferase assays. (A) The phenotypes of calluses bombarded with different configurations of *TaPpm1*s, *TaPpb1*, and the empty vector pCXSN. (B) The expression of *TaPpm1*, *TaPpb1*, and seven structural genes in calluses bombarded with *TaPpm1s* and *TaPpb1*. All structural genes were selected from unigenes of the transcriptome data with high expression in purple samples. The expression of transcription factor genes and structural genes are distinguished with black and grey bars, respectively. Expression is relative to that of *TaActin*. (C) Promoter activation assays in *Nicotiana benthamiana* leaves using a dual-luciferase assay. Firefly luciferase activity was normalized to the Renilla internal control (LUC/REN). Data are means (±SD) determined from four replicates.

To directly detect the transactivating capabilities of *TaPpm1* and *TaPpb1*, particle bombardment-mediated transient assays were performed in wheat callus (Fig. 7A). Compared with the empty vector (pCXSN), both *TaPpm1a* alone and the combination of *TaPpm1a* and *TaPpb1* (Supplementary Fig. S9) produced purple spots in the calluses, while none were observed in calluses bombarded with *TaPpm1b*, *TaPpm1c*, or *TaPpm1d*. This implied that these three mutated variants had lost their function. Calluses bombarded with *TaPpb1* had no anthocyanin accumulation, which may be attributed to the lack of a functional co-regulator MYB gene in the receptor callus.

qRT-PCR was employed to detect the expression of *TaPpm1*, *TaPpb1*, and seven anthocyanin-related structural genes in the bombarded calluses (Fig. 7B). Among the structural genes, only *CHI* and *ANS* were increased in the *TaPpm1a*-expressing callus. *ANS*, present only in the anthocyanin pathway, is an important branching-point gene that catalyses the conversion of leucoanthocyanidin to anthocyanin. *CHI* belongs to the early biosynthesis genes, but also participates in many other pathways, such as the synthesis of isoflavonoids, phlobaphenes, and flavonols. Thus, *ANS* is suggested to be the most important structural gene accounting for anthocyanin production in this system. In general, it can be proposed that TaPpm1a acts as a direct regulator involved in the activation of *ANS* and subsequent anthocyanin accumulation, while the other three TaPpm1 variants have lost this function.

### The transcriptional activation function of TaPpm1 and TaPpb1

A dual luciferase assay with three luciferase reporter constructs containing diverse promoters was used to detect the activation function of TaPpm1 (TaPpm1a and TaPpm1b) and TaPpb1 in tobacco (Fig. 7C). TFs alone failed to activate the promoter of *ANS*, while the combination of the TaPpm1a and TaPpb1 induced high luciferase activity. The *TaPpm1b* variant co-infiltrated with *TaPpb1* did not show any transcriptional activation. The different configurations of TaPpm1, TaPpb1, and empty vectors did not change the expression level of the luciferase genes driven by the *TaPpb1a*/*b* promoters. However, the luciferase driven by the *TaPpb1a* promoter displayed higher expression compared with that driven by the *TaPpb1b* promoter.

## Discussion

Wheat, as a key food resource, has recently attracted additional attention because of the health-related functions of the anthocyanins in its grain. Two genes governing the purple pericarp trait have been mapped on chromosomes 2A and 7D (Tereshchenko *et al.*, 2012), and have been further identified as a bHLH and an MYB gene, respectively (Khlestkina, 2013; Himi and Taketa, 2015). Research on model plants has revealed that the anthocyanin biosynthesis pathway is regulated by the activity of MBW complexes (Baudry *et al.*, 2004; Hichri *et al.*, 2011). Among them, WD40 proteins are more likely to enhance gene activation, while the specific expression of the bHLH or MYB components ultimately initiates the expression of target genes and regulates the accumulation of anthocyanin (Ramsay and Glover, 2005). Recent studies have implied that *TaMYC1* (bHLH, identical to *TaPpb1*) plays a role in determining the purple pericarp trait (Shoeva *et al.*, 2014; Zong *et al.*, 2017). However, whilst *TaMYC1* was necessary for conferring anthocyanin accumulation in the pericarp, it was not by itself sufficient. The precise genetic basis of, and systematic mechanism for, the formation of purple pericarps are still unknown. In this study, we provide genetic and molecular evidence to show how two TFs, TaPpm1 and TaPpb1, co-regulate anthocyanin synthesis in the pericarp.

### *TaPpm1* and *TaPpb1* collaboratively regulate the purple pericarp phenotype of wheat

Two anthocyanin regulator genes, *TaPpm1* and *TaPpb1*, which are highly expressed in the purple pericarp of the wheat variety H76, were screened-out through transcriptome and expression profile analyses (Figs 2, 3). Sequence variants of *TaPpm1* and *TaPpb1* in purple and non-purple pericarp lines were detected in the coding and promoter regions (Fig. 4). Genotyping individual plants in the F_2_ populations demonstrated that purple pericarps were associated with both *TaPpm1a* and *TaPpb1a* (Fig. 5). Moreover, we showed that the interaction of TaPpm1a with TaPpb1 positively promoted the expression of downstream structural genes, such as *ANS* (Fig. 7C). Consequently, it can be concluded that TaPpm1 combined with TaPpb1 co-regulates anthocyanin synthesis in the wheat pericarp through a MBW complex.

Early studies in other species suggested that MYB and bHLH work via formation a dimer to activate structural genes and induce pigment production (Yamagishi *et al.*, 2010; Lai *et al.*, 2016). In Y2H assays, we demonstrated that only TaPpm1a interacts strongly with TaPpb1 (Fig. 6), while the other three mutated variants of TaPpm1 did not show obvious interactions. This may be due to the frame shifts or truncated peptides caused by the insertion mutations (Supplementary Fig. S4A). A previous study revealed that sequence variations in the binding domain of MYB proteins affect their binding ability on target genes through less effective interactions with bHLH proteins (D’Amelia *et al.*, 2018). Similarly, it can be speculated that the mutation sites were critical for TaPpm1 interaction with TaPpb1, so that the mutations directly affected the activation of downstream genes and subsequent anthocyanin production.

In addition, we demonstrated that the combinations of different *TaPpm1* and *TaPpb1* variants were in accordance with pericarp colours in 34 different varieties (Fig. 4C), hinting that the regulatory mechanism is conserved among varieties. Consequently, the specific markers developed in this study for *TaPpm1* and *TaPpb1* can be used as a tool for convenient selection of purple pericarp varieties in wheat breeding programmes.

### Transcriptional regulation of the regulators TaPpm1 and TaPpb1

An integrated model for the gene regulation network that determines anthocyanin pigmentation in eudicots has been presented (Albert *et al.*, 2014; Montefiori *et al.*, 2015). Hierarchical and feedback mechanisms between the MYB and bHLH components of the MBW activation complex are important features of flavonoid regulation (Hichri *et al.*, 2011). In petunia, ectopic expression of *AN2* (R2R3-MYB activator) in leaves resulted in ectopic expression of the bHLH gene *AN1* (Spelt *et al.*, 2000). In *Medicago truncatula*, Y1H analysis suggested that the bHLH factor MtTT8 regulated its own expression as part of a positive feedback loop, through an MBW complex, that ultimately contributes to the regulation of anthocyanin synthesis (Li *et al.*, 2016). Similarly, based on the results of the Y1H assay in our present study, the combination of TaPpm1a and TaPpb1 can efficiently activate the promoter of *TaPpm1*, while either TaPpm1a or TaPpb1 alone is not able to do so (Supplementary Fig. S10). The results indicate that TaPpm1 also regulates its own expression as part of a positive feedback loop, through an MBW complex.

Moreover, we observed that *TaPpm1* transcripts increased by a factor of 31.6 times when the seeds were exposed to strong light (Fig. 2B); this increase was associated with abundant colour accumulation in purple pericarps (Fig. 1). Consequently, we propose that *TaPpm1* is induced by the light stimulus. Its promoter region contains 21 light-response elements, including G- and ACE-boxes (Supplementary Table S7). It has been demonstrated that the bZIP protein HY5 can bind to the MYB activator through these elements, and subsequently initiate light response-dependent anthocyanin accumulation (Shin *et al.*, 2013). A homologous HY5, found in our transcriptome data, was significantly up-regulated by light stimulus. It would be interesting to identify the regulatory relationship between HY5 and TaPpm1 in high-light conditions.

The coding region of *TaPpb1* was identical in different lines, while the promoter region displayed two different variants (Fig. 4). There were six repeats in the promoter of *TaPpb1a*, and only one repeat unit in *TaPpb1b*. Previous studies reported that the repeat units in the promoters of *MdMYB10* in apple and of *RLC1* in cotton worked as enhancers (Espley *et al.*, 2009; Gao *et al.*, 2013). Similarly, the dual-luciferase assay showed that the promoter of *TaPpb1a* induced higher expression of the fused genes compared with that driven by the *TaPpb1b* promoter (Fig. 7C). Hence, we speculate that some endogenous upstream factors may bind to the replicated 261-bp units in the promoter of *TaPpb1a* and enhance its expression level, subsequently resulting in production of sufficient TaPpb1 combined with TaPpm1a to promote anthocyanin synthesis in the purple pericarp. 

### The spatial expression of *TaPpb1* and *TaPpm1*

It has been shown that the production of anthocyanins in different tissues is often regulated by tissue-specific expression of bHLH or MYB proteins (Yamagishi *et al.*, 2010; Yuan *et al.*, 2014). During seed development in the present study, the expression level of *TaPpb1* gradually increased and reached its maximum at 22 DAP (Fig. 3B), followed by a slight decrease. Among the different tissues examined, *TaPpb1* presented significant expression in seeds (Fig. 3A). Combining this finding with the five seed-specific *cis*-elements existing in the promoter region of *TaPpb1* (Supplementary Table S7), it suggests that *TaPpb1* is a seed-specific expression gene. This is consistent with previous results that found that the *Pp3* locus, where *TaPpb1* is located, specifically participates in anthocyanin synthesis in the pericarp (Khlestkina, 2013). In a recent study, it was shown that a homologous bHLH *AetMYC* on chromosome 2D modulated anthocyanin synthesis in the coleoptile of *Aegilops tauschii*, the diploid ancestor of the wheat D genome (Cao *et al.*, 2017). Similar cases were found in maize; for example, the different *b* alleles (orthologous to *TaPpb1*), *B-peru* and *B-I*, that regulate specific anthocyanin production in seeds and vegetative tissues, respectively (Selinger and Chandler, 1999). Thus, we propose that different bHLH homologues may participate in anthocyanin synthesis in wheat in a tissue-specific manner.

In previous studies, *Rc-D1* (also termed *TaPpm1* in our study) was suggested as the candidate gene participating in anthocyanin synthesis in wheat coleoptiles (Himi and Taketa, 2015; Wang *et al.*, 2016). Likewise, we found that *TaPpm1* was also highly expressed in coleoptiles, and our genetic analysis revealed that the genotype of *TaPpm1* was co-segregated with the coleoptile colours in progenies of ‘A14×H76’ (Fig. 2A; Supplementary Fig. S11). In addition, we observed that TaPpm1a alone induced anthocyanin synthesis in calluses, which may be caused by the impure background of wheat where TaPpm1 co-works with other bHLH TFs expressed in the callus (Fig. 7A). It can be concluded that TaPpm1, co-working with bHLH TFs, not only plays a role in anthocyanin synthesis in seeds, but also in other tissues, such as coleoptiles and calluses. A similar mechanism has also been shown in potato, where that the allelic configuration of different loci, like the bHLHs, together with the widely expressed MYB genes are responsible for the co-regulation of anthocyanin distribution in tissues (Jung *et al.*, 2009; Liu *et al.*, 2016).

In conclusion, two transcription factors, TaPpm1 and TaPpb1, were identified in wheat and shown to co-regulate the pericarp colour through formation of a complex. The allelic variations of *TaPpm1* affect anthocyanin pigmentation by altering the binding ability with bHLH, whereas variation in the *TaPpb1* promoter changes its expression level. TaPpm1 and TaPpb1 have different spatial expression profiles. *TaPpm1* is widely expressed in many tissues, while *TaPpb1* is specifically expressed in seeds. These findings reveal the molecular and genetic basis of anthocyanin production in the wheat pericarp and provide clues that will facilitate further study of the sophisticated anthocyanin biosynthesis network in different tissues of wheat.

## Supplementary data

Supplementary data are available at *JXB* online.

Fig. S1. Expression analysis of anthocyanin structural genes.

Fig. S2. Amplification of the genetic sequences and promoter regions of *TaPpm1* and *TaPpb1* in 12 different-coloured wheat lines.

Fig. S3. Structure of mutations in the variants of *TaPpm1* and *TaPpb1*.

Fig. S4. Protein sequence alignments of MYBs and bHLHs.

Fig. S5. Deduced structure of the insertion region in the promoter of *TaPpb1a*.

Fig. S6. Comparative mapping of *TaPpm1* and *TaPpb1*.

Fig. S7. Subcellular localization of the TaPpm1 and TaPpb1 proteins in onion epidermal cells.

Fig. S8. Yeast two-hybrid autoactivation analysis of TaPpb1 and TaPpm1.

Fig. S9. Transient assay of the *TaPpm1* and *TaPpb1* complex in wheat calluses.

Fig. S10. The complex of TaPpm1 and TaPpb1 activates the promoter of *TaPpm1*.

Fig. S11. Association analysis between coleoptile colours and the genotypes of *TaPpm1* and *TaPpb1* in F_3_ individuals.

Table S1. Primers used in this study.

Table S2. Inheritance pattern of pericarp colours in the F_2_ progenies of the cross between the white-pericarp wheat ‘A14’ and the purple-pericarp wheat ‘H76’.

Table S3. Summary of the read data for characteristics of the three wheat pericarp libraries.

Table S4. Unigenes that were differently expressed in purple samples (H-17d and H-10p) compared with those in non-purple samples (H-10d).

Table S5. Structural genes related to anthocyanin biosynthesis.

Table S6. Sequences of MYB proteins and bHLH motifs used in the phylogenetic tree.

Table S7. Conserved elements in the promoters of *TaPpm1* and *TaPpb1*.

Supplementary Figure S1-S11

Supplementary Table S1 S2 S3 S7

Supplementary Table S4

Supplementary Table S5

Supplementary Table S6
