# Supplementary material for: Two transcription factors TaPpm1 and TaPpb1 co-regulate anthocyanin biosynthesis in purple pericarps of wheat
Source: J Exp Bot. 2018 Mar 17;69(10):2555–67. doi: 10.1093/jxb/ery101 (PMC5920291; doi:10.1093/jxb/ery101)
Supplement: Supplementary Figure S1-S11 [file ery101_suppl_supplementary_figure_s1-s11.pdf]

Two transcription factors TaPpm1 and TaPpb1 co-regulate the anthocyanin biosynthesis in purple pericarp of wheat

Wenhui Jiang, Tianxiang Liu, Wenzhi Nan, Diddugodage Chamila Jeewani, Yanlu Niu, Chunlian Li, Yong Wang, Xue Shi, Cong Wang, Jiahuan Wang, Yang Li, Xin Gao*, Zhonghua Wang*

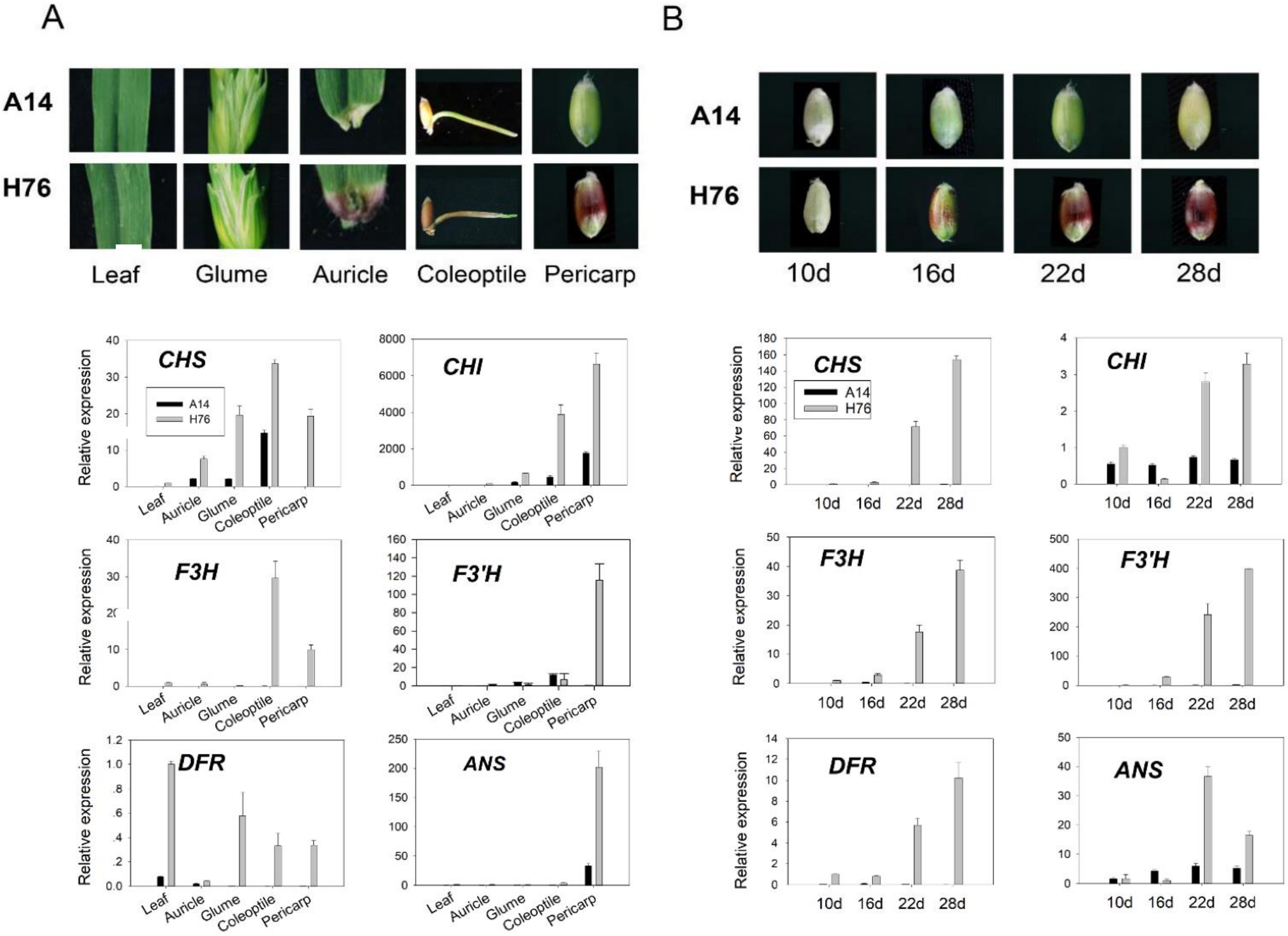

Supplementary Figure S1. Expression analysis of anthocyanin structural genes. (A) Relative expressions of structural genes in different coloured tissues of the two wheat lines: A14 (white) and H76 (purple). (B) Relative expression of structural genes at four physiological stages during pericarp development. The data are expressed as the mean ± SD of three biological replicates.

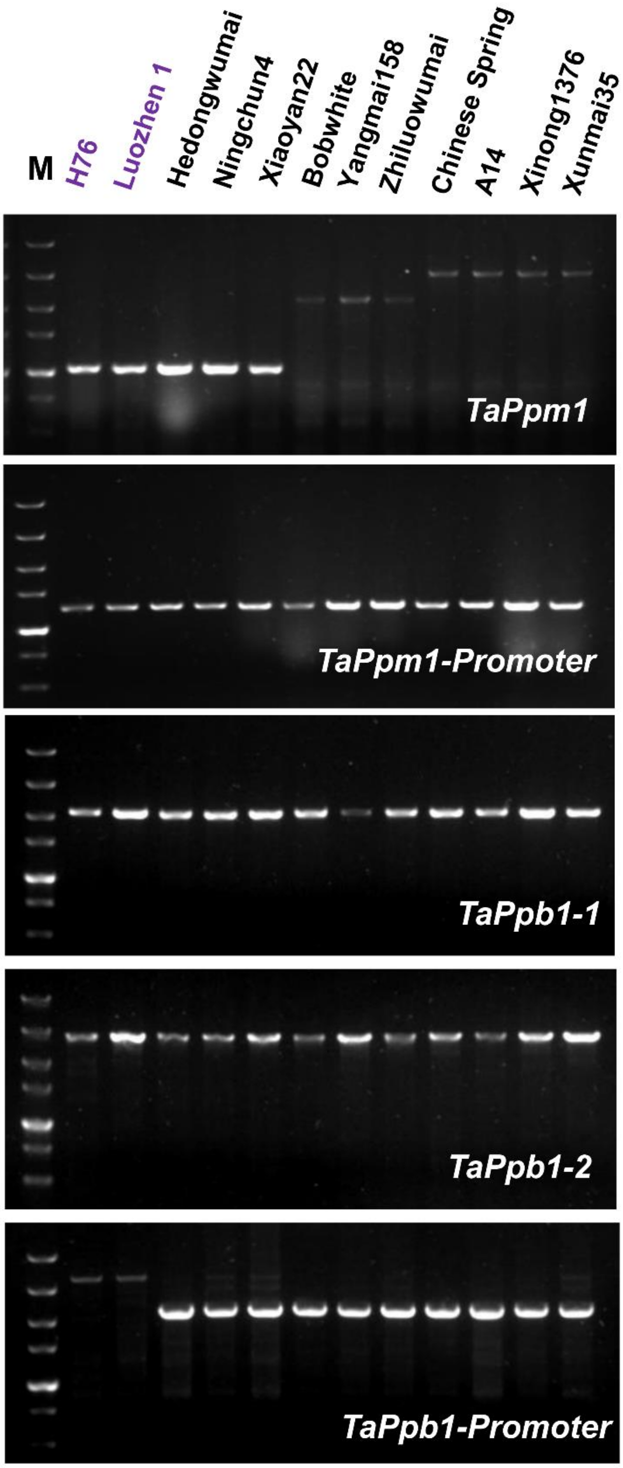

Supplementary Figure S2. Amplification of the genetic sequences and promoter regions of *TaPpm1* and *TaPpb1* in 12 of *TaPpm1* is amplified with primer set 7D01. Two primer sets 2A01a and 2A01b were dedifferent-coloured wheat lines. Varieties marked in purple have purple pericarp phenotypes (H76 and Luozhen 1). The genomic sequence signed for acquiring 5' portion (*TaPpb1-1*) and 3' portion (*TaPpb1-2*) of *TaPpb1* as it is too long to be amplified in one reaction. The promoter regions of *TaPpm1* (*TaPpm1-Promoter*) and *TaPpb1* (*TaPpb1-Promoter*) were amplified using primer sets 7DPRO1 and 2APRO1.

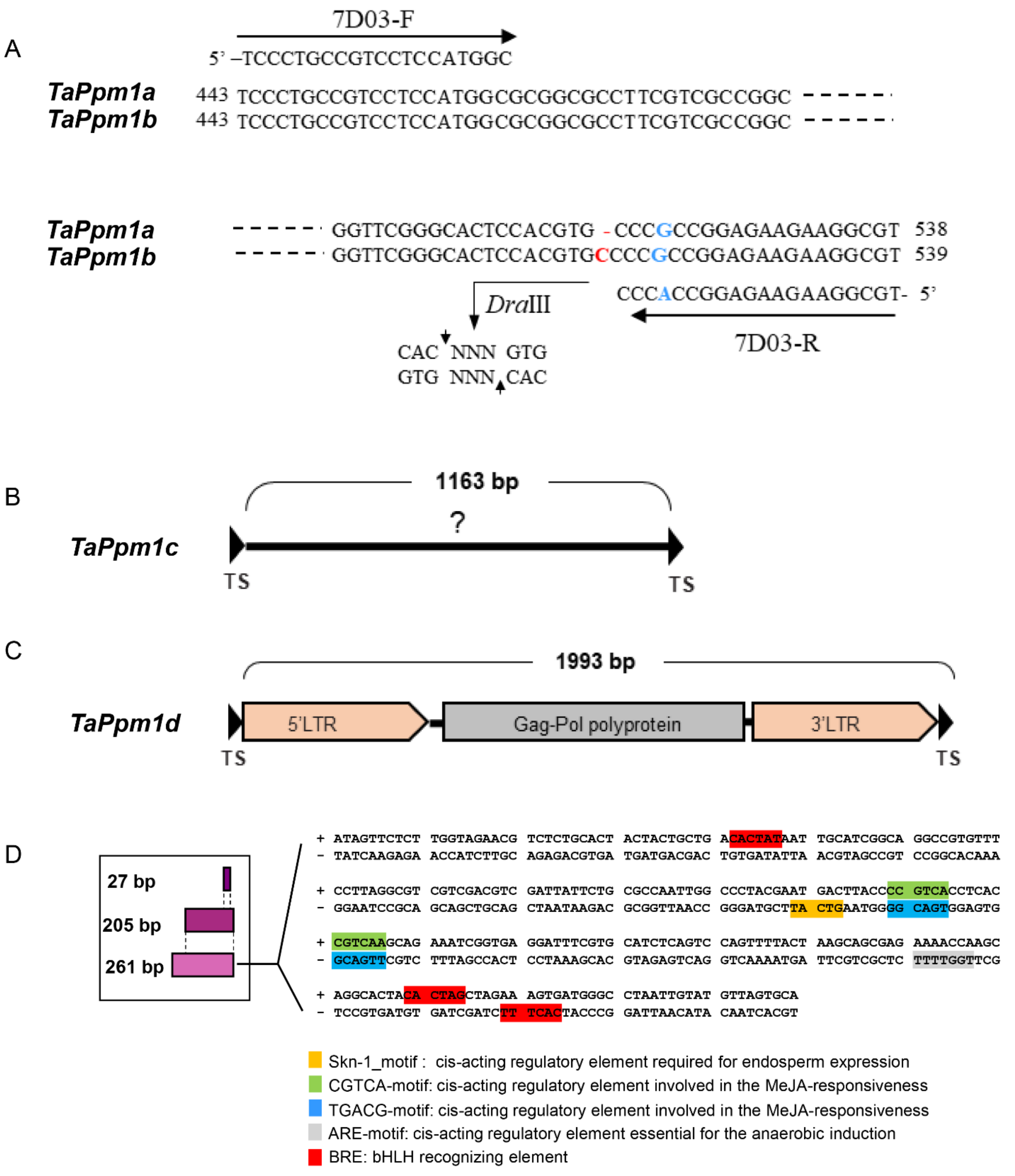

Supplementary Figure S3. Structure of mutations in the variants of *TaPpm1* and *TaPpb1*. (A) Partial alignments of *TaPpm1a* and *TaPpm1b*. Primers used for PCR are shown with black arrows. The 7D03-R primer generates a recognition site of *Dra*III (CACNNNGTG) in the fragment of *TaPpm1b* with 1-bp insertion, compared with that of *TaPpm1a*. (B) The insertion sequence of *TaPpm1c* has duplicated target sites. (C) The insertion sequence of *TaPpm1d* is a typical *Copia*-like LTR retrotransposon. Bold orange arrows represent retrotransposon LTRs; bold gray bar represent open reading frame encoding functional Gag-Pol polyprotein and bold black triangles represent duplicated target sites. (D) The characteristics of the specific 261-bp insertion sequence in *TaPpb1*. The 261-bp unit possesses five kinds of motifs: Skn-1 motif, CGTCA-motif, TGACG-motif, ARE motif and BRE motif.

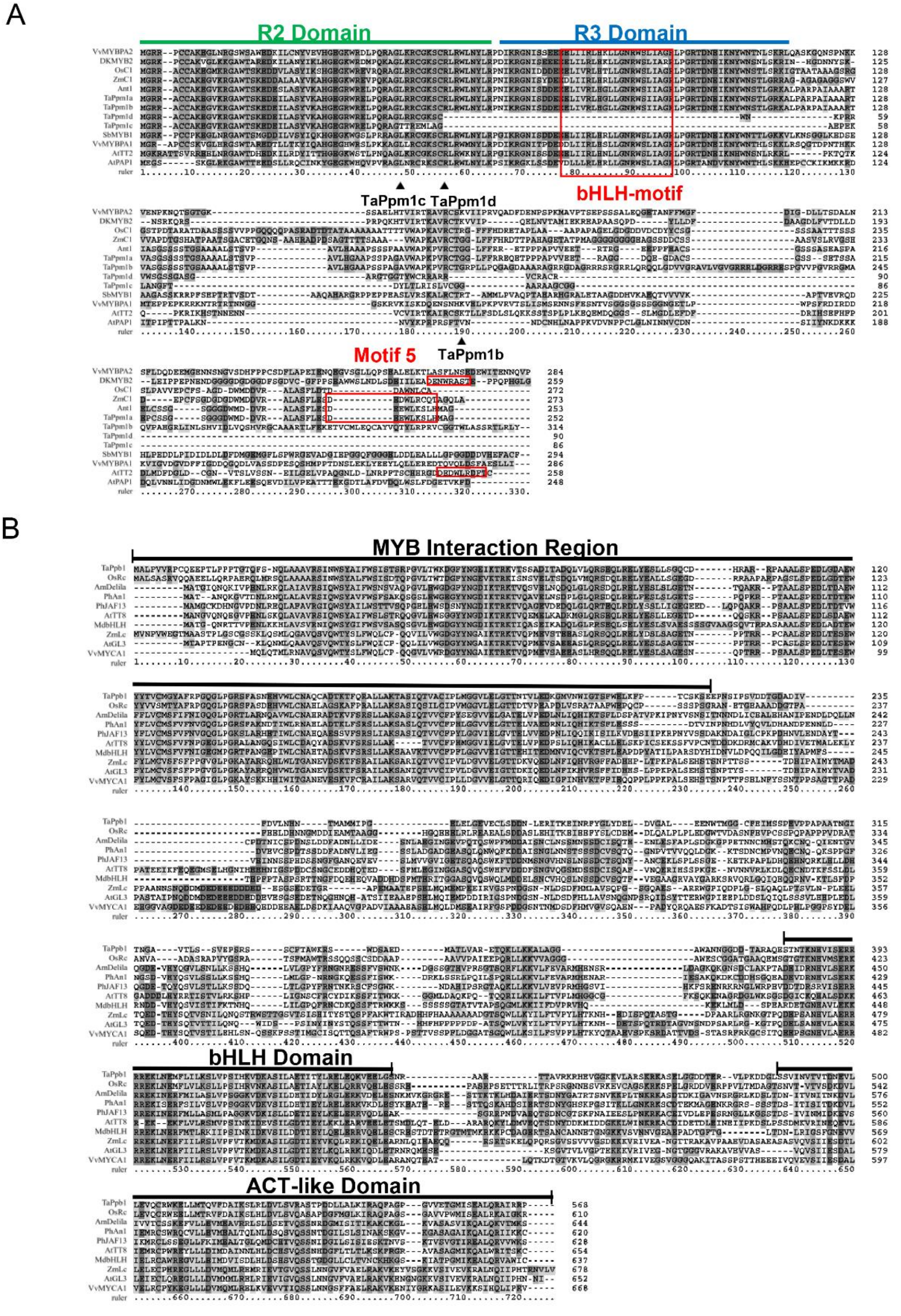

Supplementary Figure S4. Protein sequence alignments of MYBs and bHLHs. (A) Alignment between the four TaPpm1 proteins (TaPpm1a, TaPpm1b, TaPpm1c and TaPpm1d) and anthocyanin-related R2R3-MYBs in other species. Black arrows represent the mutated sites of allelic variants (TaPpm1b, TaPpm1c, TaPpm1d), compared with TaPpm1a. (B) Alignment between TaPpb1 and other anthocyanin-related bHLH proteins. MYB interaction region, bHLH domain and ACT-like domain are conserved among the bHLH transcription factors.

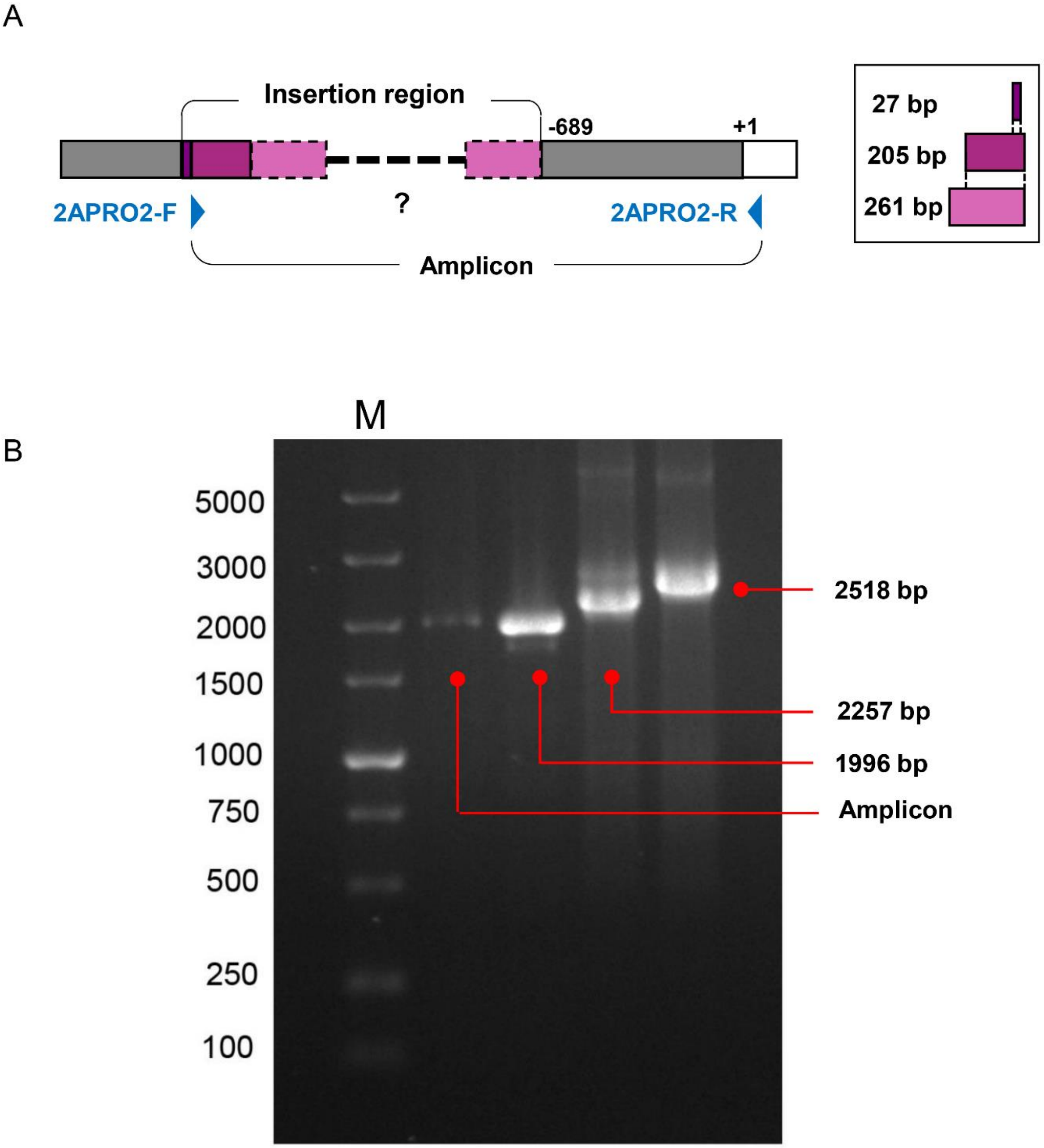

Supplementary Figure S5. Deduced structure of the insertion region in the promoter of *TaPpb1a*. (A) The structure of the promoter region in *TaPpb1a*. Grey rectangle represents the promoter region and two dark purple rectangles indicate the specific sequence of insertion region which has been sequenced. Since the insertion region is too long and contains several tandem repeats, it cannot be assembled by paired-end sequencing and the number of repeat is unknown. The dotted purple boxes and dotted lines represent the unknow number of the 261-bp unit. 2APRO2 primer set was used to amplify the insertion sequence. (B) Three sequences with known lengths were used to estimate the length of insertion region and the repeat number in the insertion fragment. The length of amplicon with four, five or six repeats in insertion fragments were corresponding to 1996 bp, 2257 bp or 2518 bp, respectively. It shows that the amplicon has the same length with the 1996-bp fragment, indicating that there are four 261-bp repeat units in the insertion region.

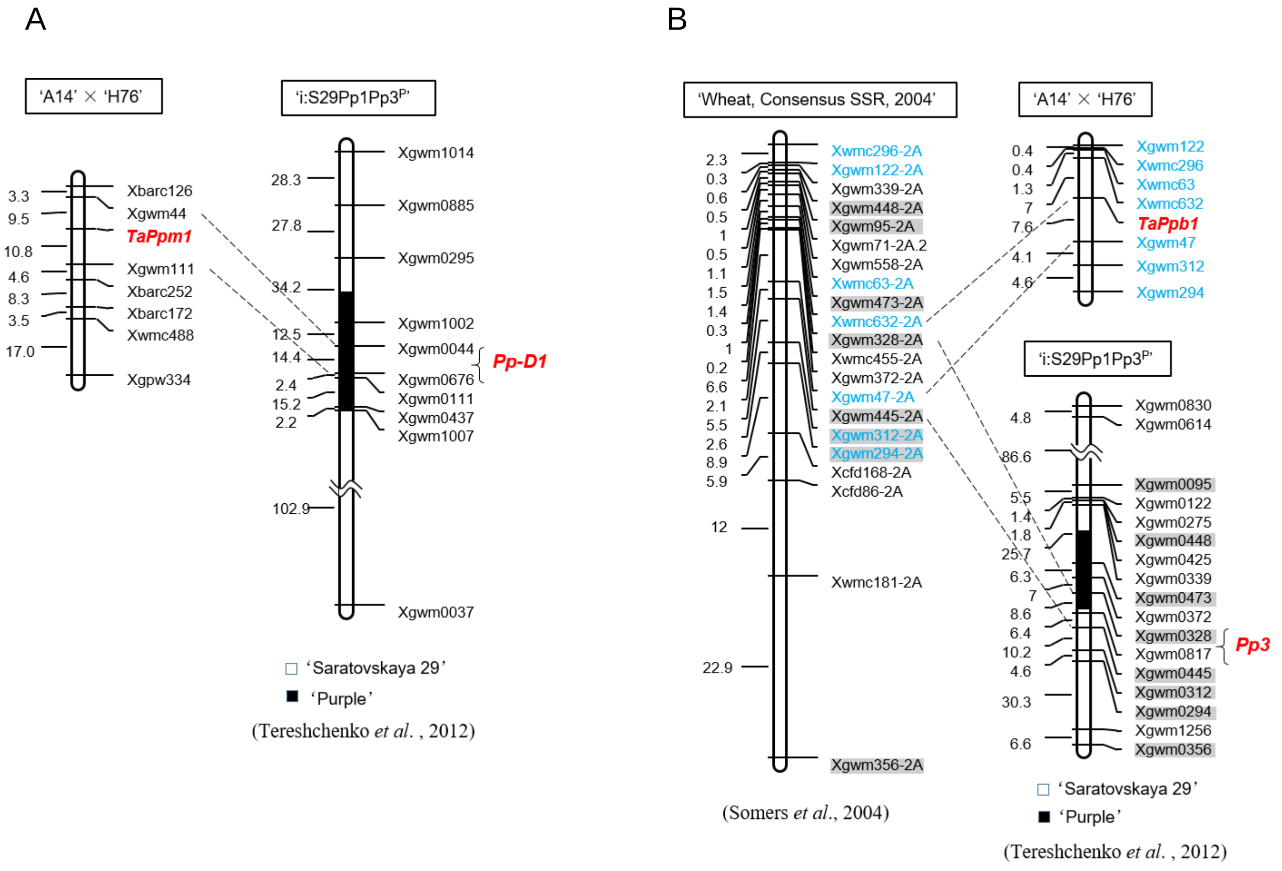

Supplementary Figure S6. Comparative mapping of *TaPpm1* and *TaPpb1*. (A) The comparative map between *TaPpm1* and *Pp-D1* on chromosome 7D. (B) The comparative map between *TaPpb1* and *Pp3* on chromosome 2A. Markers with gray background in 'i:S29Pp1Pp3^{P}' map and makers with blue words in our 'A14× H76' map can be found in media map 'Wheat, Consensus SSR, 2004'.

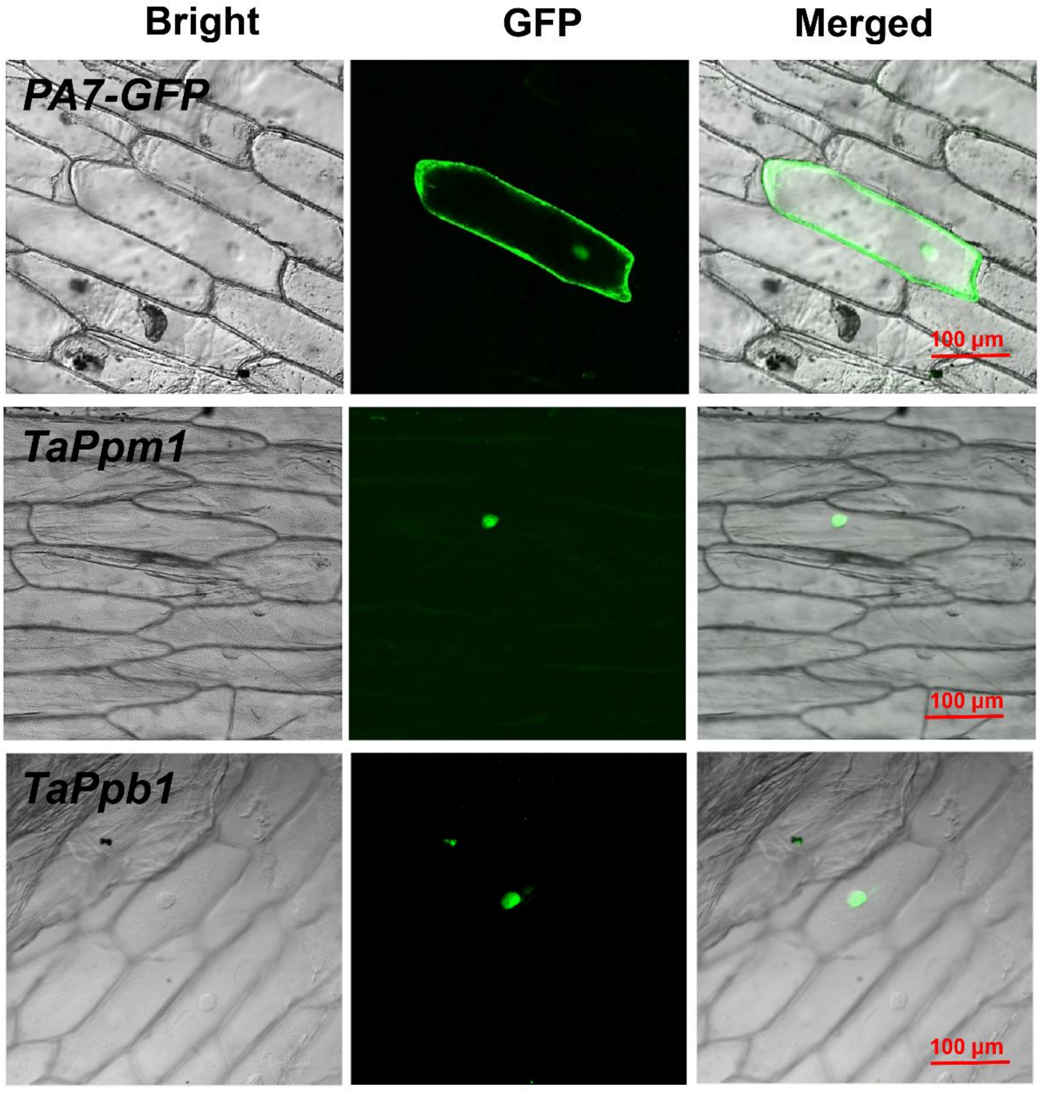

Supplementary Figure S7. Subcellular localization of the TaPpm1 and TaPpb1 protein in onion epidermal cells. The TaPpm1-GFP and TaPpb1-GFP fusion proteins were localized to the nucleus.

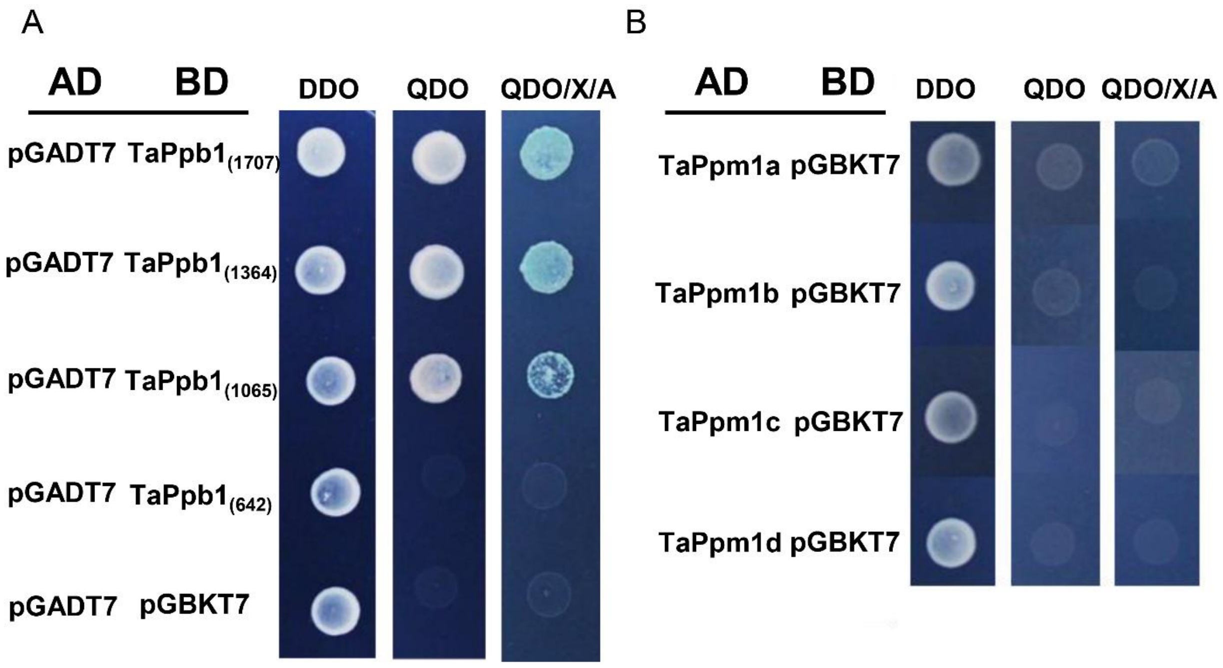

Supplementary Figure S8. Yeast two-hybrid autoactivation analysis of TaPpb1 and TaPpm1. (A) The autoactivation analysis of full length TaPpb1 ($TaPpb1_{(1707)}$) and relevant truncated proteins translated by partial sequences of *TaPpb1* (1-1364, 1-1065 and 1-642 bp). (B) The autoactivation analysis of the four variants of TaPpm1.

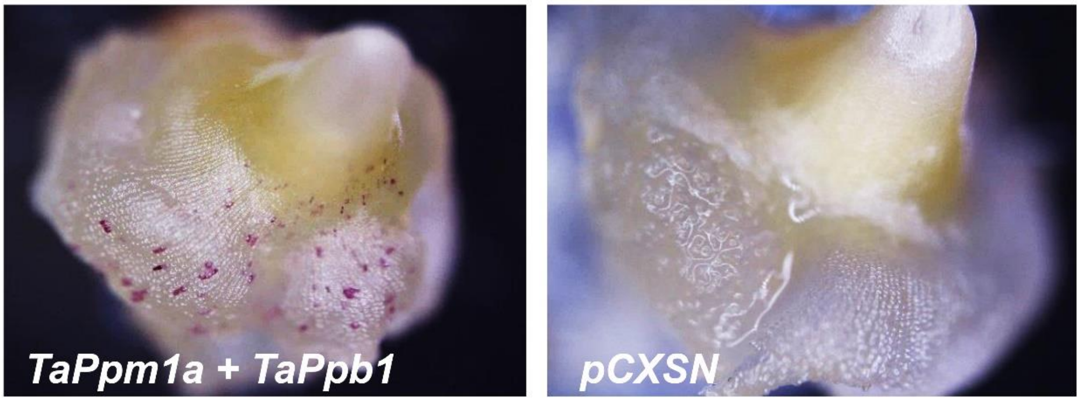

Supplementary Figure S9. Transient assay of the *TaPpm1* and *TaPpb1* complex in wheat calluses. The mix of expression constructs of *TaPpm1* and *TaPpb1* were delivered into the wheat calluses of the white-grained wheat Chinese Spring with particle bombardment.

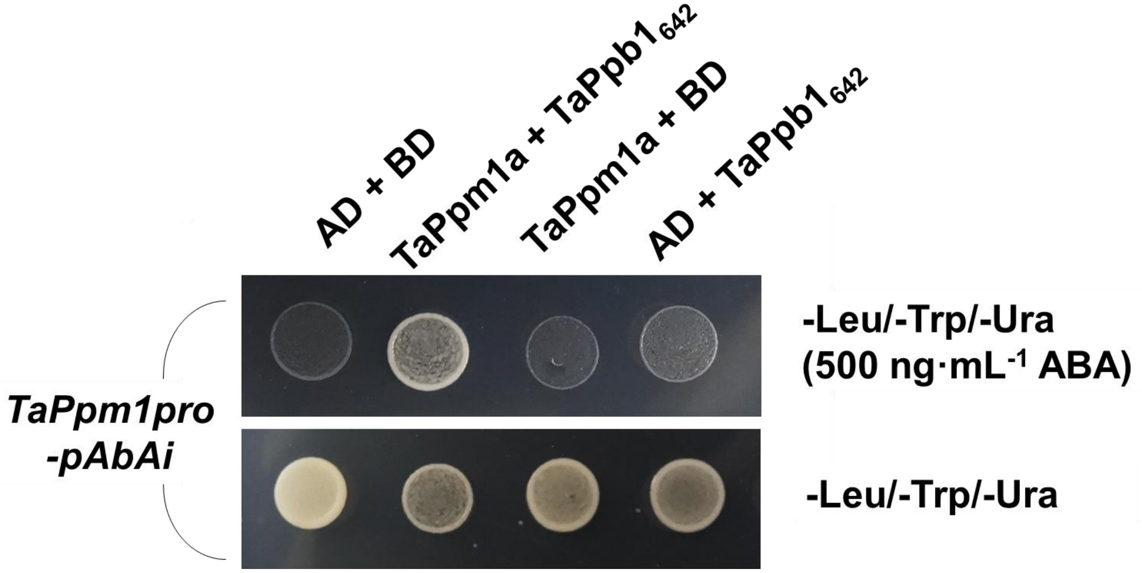

Supplementary Figure S10. The complex of TaPpm1 and TaPpb1 activates the promoter of *TaPpm1*. The combinations of transcription factors with empty vectors activate *TaPpm1* promoter in the Y1H assay. Growth was recorded in the presence or absence of 500 ng·mL^{-1} aureobasidin A.

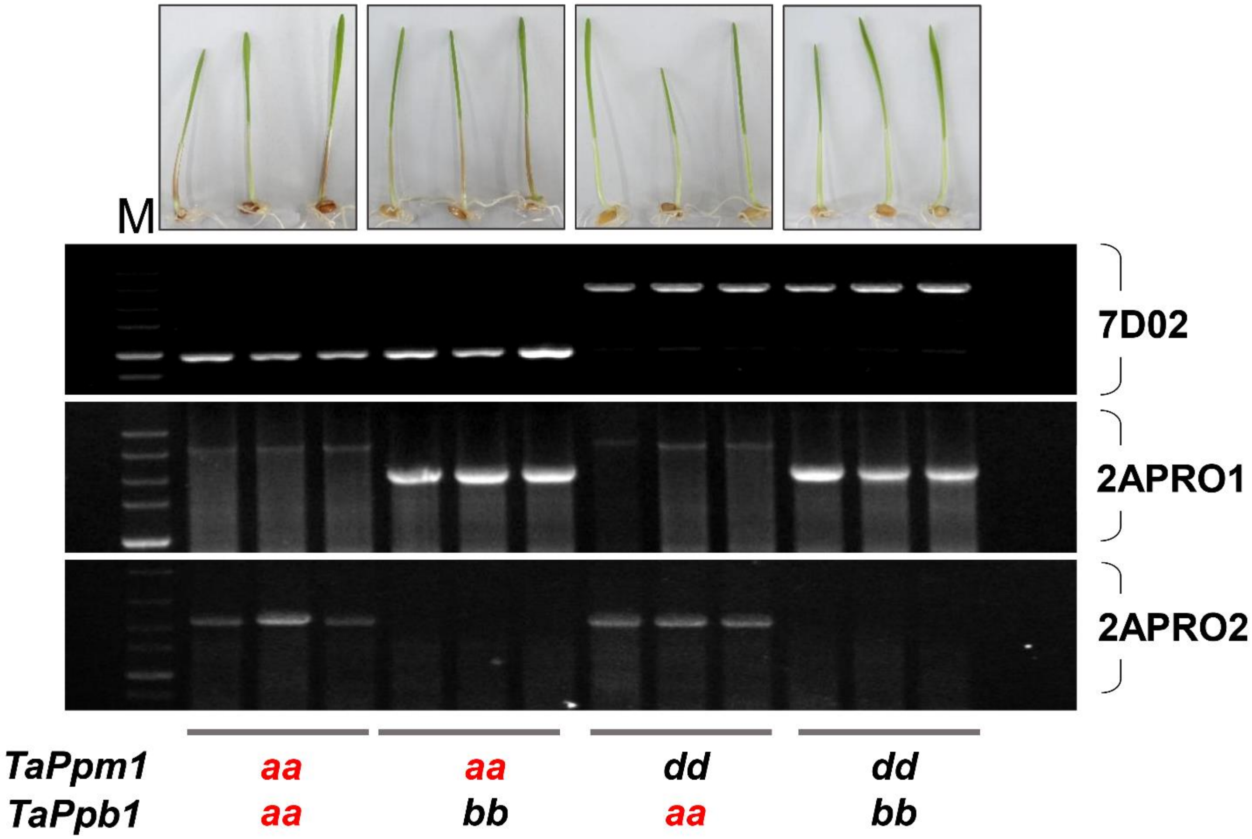

Supplementary Figure S11. Association analysis between coleoptile colours and the genotypes of *TaPpm1* and *TaPpb1* in F_3 individuals. We have detected the coleoptile colours and *TapPm1* and *TaPpb1* genotypes of 96 F_3 progenies, derived from selfing F_2 plants. The colour variation in coleoptiles is co-separated with genotypes of *TaPpm1*, but unrelated to the genotypes of *TaPpb1*.
