# Supplementary material for: Two transcription factors TaPpm1 and TaPpb1 co-regulate anthocyanin biosynthesis in purple pericarps of wheat
Source: J Exp Bot. 2018 Mar 17;69(10):2555–67. doi: 10.1093/jxb/ery101 (PMC5920291; doi:10.1093/jxb/ery101)
Supplement: Supplementary Table S1 S2 S3 S7 [file ery101_suppl_supplementary_table_s1_s2_s3_s7.pdf]

Two transcription factors TaPpm1 and TaPpb1 co-regulate the anthocyanin biosynthesis in purple pericarp of wheat

Wenhui Jiang, Tianxiang Liu, Wenzhi Nan, Diddugodage Chamila Jeewani, Yanlu Niu, Chunlian Li, Yong Wang, Xue Shi, Cong Wang, Jiahuan Wang, Yang Li, Xin Gao*, Zhonghua Wang*

Supplementary Table S1. Primers used in this study

Name	Forward (5'-3')	Reverse (5'-3')
**Primers designed for qRT-PCR assay**		
TaActin	TGTTGTTCTCAGTGGAGGTTCT	CTGTATTTCCTTTCAGGTGGTG
TaPpm1-RT	GGTTGAGAGAGGGAGAGGGGAT	CGCAGACCAGCTCGCTGC
TaPpm2-RT	GAGGACGTGCAGCGCGAA	CGCAGACCGGCTTTGAGG
TaPpm3-RT	CGCTCACTCGCTCTCTAACACAA	ACCCCTTCCTTGGCACTCC
TaPpb1-RT	CGGCCTGAGCAGTGTCATCA	GTGTCACGCTTTCAACAACATCC
CHS-RT	TGGAAAACAACCTACTACATACAGC	CAGACTAACAGGAGATCGAATGCAT
CHI-RT	CGTCCTCGTATTTGTCCGCTG	GAACCATAGTCACATATCACGAGG
F3H-RT	TGATTGATGCGTGGTGGGA	CAGAAACCAGTACGAAATATACGC
F3'H-RT	TAGCTCTCCAGTCTTCCTTGCC	GCCGTCGGCGTACACGA
F3'5'H-RT	GGGCTAGGATCAGATCAACTCG	AATACAAAACAGTACGCACAGAAAAC
DFR-RT	GCTCATCACAGGGAATGAAGC	GTCGTCGACGCCTGCGA
ANS-RT	CTTCTCGGCGCCGTCCG	GTCAACCGCCAGGCCGT
**Primers designed to amplify gene sequences and promoter region**		
7D01	CGAGATAGAGATAGGGAGAGGGGTT	TGAACAACTCAGTACAGTCGTAGTTTATA
7D02	GCTGGTACTACTCGCGAGATGTTAG	TGAACAACTCAGTACAGTCGTAGTTTATA
7D03	TCCCTGCCGTCCTCCATGGC	TGCGGAAGAAGAGGCCACCC
7DPRO1	CACGACAACGTGAAAAATTATCCT	ATCCCCTCTCCCTCTCTCAACCC
2A01	AACAGAGCAAAGAGATTTGACTAAA	ATAGCCACCAAATTTACGAATCTAC
2A01a	AACAGAGCAAAGAGATTTGACTAAA	ACTAAATGTCACTCAACAGGGACTT
2A01b	CGACTGAGACATAAAAGTCCCTG	ATAGCCACCAAATTTACGAATCTAC
2APRO1	AAGAAATGACACTTCGCCACAA	GGTGGGCTCTTCCTGGCAT
2APRO2	GTGATGGGCCTAATTGTATCGG	GGTGGGCTCTTCCTGGCAT
**SSR markers used in this study**		
Xbarc126	CCATTGAAACCGGATTTGAGTCG	CGTTCCATCCGAAATCAGCAC
Xbarc172	GCGAAATGTGATGGGGTTTATCTA	GCGATTTGATTTAACTTTAGCAGTGAG
Xbarc252	CGCAAAAACATGAACACTCTTAAAACT	CGCAAAAATGTTCGTGTAACTCTAAAA
Xgpw334	GGATGGACTGGGCAGGAAT	AGCACTGACTTTGCCTTAACT
Xgwm111	TCTGTAGGCTCTCTCCGACTG	ACCTGATCAGATCCCACTCG
Xgwm44	GTTGAGCTTTTCAGTTCGGC	ACTGGCATCCACTGAGCTG
Xwmc488	AAAGCACAACCAGTTATGCCAC	GAACCATAGTCACATATCACGAGG
Xgwm122	GGGTGGGAGAAAGGAGATG	AAACCATCCTCCATCCTGG
Xwmc296	GAATCTCATCTTCCCTTGCCAC	ATGGAGGGGTATAAAGACAGCG
Xwmc63	GTGCTCTGGAAACCTTCTACGA	CAGTAGTTTAGCCTTGGTGTGA
Xwmc632	GTTTGATTGGTCGTTCCTGGTC	AACAGCGAATGGAGGGCTTTAG
Xgwm47	TTGCTACCATGCATGACCAT	TTCACCTCGATTGAGGTCCT
Xgwm312	ATCGCATGATGCACGTAGAG	ACATGCATGCCTACCTAATGG
Xgwm294	GGATTGGAGTTAAGAGAGAACCG	GCAGAGTGATCAATGCCAGA
**Primers designed to construct various vectors**		
Ppm1-PCXSN	GAGAGGGGATGGGGAGGA	GTGAGAGGTTAATTAACCGGCCA
Ppm1b-PCXSN	GAGAGGGGATGGGGAGGA	TCAGTACAGTCGTAACGTA
Ppb1-PCXSN	AACAGAGCAAAGAGATTTGACTAAA	AACAGAGCAAAGAGATTTGACTAAA
Ppb1-BK1	ATGGCCATGGAGGCCGAATTCATGGCGCTGCCAGTAGTTC	TCGACGGATCCCCGGGAATTCTCATGGCCTGCGAATAGCT
Ppb1-BK2	ATGGCCATGGAGGCCGAATTCATGGCGCTGCCAGTAGTTC	TCGACGGATCCCCGGGAATTCACTTCATGGCGTTTTCTGACAG
Ppb1-BK3	ATGGCCATGGAGGCCGAATTCATGGCGCTGCCAGTAGTTC	TCGACGGATCCCCGGGAATTCCTGTGTCTCCCTGGCGACC
Ppb1-BK4	ATGGCCATGGAGGCCGAATTCATGGCGCTGCCAGTAGTTC	TCGACGGATCCCCGGGAATTCTCACGATGTTGGGAACTTCAGCTC
Ppm1-AD	GCCATGGAGGCCAGTGAATTCATGGGGAGGAGGGCGTG	ATGCCCACCCGGGTGGAATTCTTAACCGGCCATGTGCAG
Ppm1b-AD	GCCATGGAGGCCAGTGAATTCATGGGGAGGAGGGCGTG	ATGCCCACCCGGGTGGAATTCTCAGTACAGTCGTAACGTA
Ppm1-pAbAi	GATGAATTGAAAAGCTTATCTCACGGATATAACATCGACCG	AGATCCCCGGGTACCGAGCTCAGGCTTCCATGTGTGCATGAACA
Ppm1a-62sk	CGCTCTAGAACTAGTGGATCCATGGGGAGGAGGGCGTG	GTCGACGGTATCGATAAGCTTTTAACCGGCCATGTGCAG
Ppm1b-62sk	CGCTCTAGAACTAGTGGATCCATGGGGAGGAGGGCGTG	GTCGACGGTATCGATAAGCTTTCAGTACAGTCGTAACGTA
Ppb1-62sk	CGCTCTAGAACTAGTGGATCCATGGCGCTGCCAGTAGTTC	GTCGACGGTATCGATAAGCTTTCATGGCCTGCGAATAGC
Ppm1-0800	GTCGACGGTATCGATAAGCTTGAAGCTAACGTGTGTATTTGTATG	CGCTCTAGAACTAGTGGATCCCCCCTCTCCCTCTCTCAACC
Ppb1-0800	GTCGACGGTATCGATAAGCTTGGCAGACATACATTTGATTTGATG	CGCTCTAGAACTAGTGGATCCACCACACTATTTCCTTCCTTCATAT
ANS-0800	GTCGACGGTATCGATAAGCTTGGAGGCACTGGTAGTGGGACT	CGCTCTAGAACTAGTGGATCCCCTCCTCCGATGGTTTGCTC
Ppm1-GFP	CGCCATGGTCGACGTACTAGTGATGGGGAGGAGGGCGT	CGCCATGGTCGACGTACTAGTGATGGGGAGGAGGGCGT
Ppb1-GFP	CGCCATGGTCGACGTACTAGTGATGGCGCTGCCAGTAGTT	CCCTTGCTCACCATCACTAGTACTGGCCTGCGAATAGCTCT

The primers prefixed with 'Ppm1' are used to amplify *TaPpm1a*, *TaPpm1c* and *TaPpm1d*, while those prefixed with 'Ppm1b' are used for *TaPpm1b* gene. The primers prefixed with 'Ppb1' are designed for *TaPpb1* gene. Primers suffixed by 'PCXSN' are used to construct plant expression vector pCXSN; Primers suffixed with 'BK', 'AD' and 'pAbAi', used in Y2H or Y1H assay, are engaged to construct pGBKT7, pGADT7 and pAbAi vectors,

respectively; primers suffixed with ‘62sk’ and ‘0800’ are used in dual luciferase assay to construct pGreenII 62SK and pGreenII 0800; primers suffixed with ‘GFP’ participate in subcellular location assay for fusing genes to PA7-GFP vector.

Supplementary Table S2. Inheritance pattern of pericarp colours in the F_2 progenies of the cross between the white pericarp wheat 'A14' and the purple-pericarp wheat 'H76'

Cross	Purple seeds		White seeds		Expected segregation ratio	χ^2	P-value
	Observed value	Theoretical value	Observed value	Theoretical value			
A14 × H76	68	71	58	55	9:7	0.310	0.05

Supplementary Table S3. Summary of the read data for characteristics of the three wheat pericarp libraries

Sample	Raw Reads Number	Raw Bases Number	Raw Reads Length (bp)	Clean Reads Number	Clean Bases Number	Clean Reads Rate (%)	Low-quality Reads Number	Total potential transcripts	Total potential genes	Average potential transcripts
H-10d	70,002,026	8,750,253,250	125	48,856,084	6,107,010,500	69.79	16,927,928			
H-10p	40,631,776	5,078,972,000	125	27,171,616	3,396,452,000	66.87	11,391,452	174,267	113,684	713.8
H-17d	45,603,786	5,700,473,250	125	30,591,398	3,823,924,750	67.08	12,770,188			

H-10d, pericarps at 10 days after pollination (DAP) without anthocyanin accumulation. H-10p, pericarps at 10 DAP induced by high light for 12 h for anthocyanin accumulation. H-17d, pericarps at 17 DAP with natural anthocyanin accumulation.

Supplementary Table S7. Conserved elements in the promoters of *TaPpm1* and *TaPpb1*

Category	Description	*Cis*-acting element	Sequence	Distance from start codon (ATG)
**Conserved elements in the promoter of *TaPpm1***				
Light	part of a light responsive element	TCCC-motif	TCTCCCT	-11, -29
	part of a light responsive element	I-box	GATAGGG	+32
	part of a light responsive element	box II	TCCACGTGGC	-366
	part of a light responsive element	chs-CMA2a	TCACTTGA	-533, -713
	light responsive element	GT1-motif	GGTTAA	-728, -584
	light responsive element	MNF1	GTGCCC(A/T)(A/T)	+773
	light responsive element	Sp1	GGGCGG	-938
	cis-acting element involved in light responsiveness	ACE	ACTACGTTGG	-813
	cis-acting element involved in light responsiveness	G-Box	CACGTT/CACGTA/CACGAC/TACGTG/TACGTG/CACATGG/GCCACGTGGA	-133, +133, +161, -161，+226, +366, -790, -828, +834, +877
Grain	*cis*-acting regulatory element required for endosperm expression	Skn-1_motif	ACGTGGC/TACGTG	-317
Hormone	*cis*-acting element involved in the abscisic acid responsiveness	ABRE	GGTCCAT	-135, -161
	cis-acting regulatory element involved in auxin responsiveness	AuxRR-core	TGACG	+199
	gibberellin-responsive element	P-box	CCTTTTG	+205
Development	*cis*-acting regulatory element related to meristem expression	CAT-box	TGGTTT	+73, +366
	cis-acting regulatory element related to meristem specific activation	CCGTCC-box	CCGTCC	+254, -303
	cis-acting regulatory element related to meristem specific activation	dOCT	CACGGATC	+664
MYB binding	MYB-recognizing element	MRE	ANCNNCC	+85, +88, +252, -260, +444, +844, +933
bHLH binding	bHLH-recognizing element	BRE	CACN(A/C/T)(G/T)	+42, -140, +220, +358, -419, +871
**Conserved elements in the promoter of *TaPpb1***				
Light	part of a module for light response	AE-box	AGAAACTT	-227
	light responsive element	Sp1	CC(G/A)CCC	+307
	cis-acting element involved in light responsiveness	ACE	GACACGTATG	-896
	cis-acting element involved in light responsiveness	G-box	CACGTA/TACGTG	+725
	light responsive element	GT1-motif	ATGGTGGTTGG	-847
Grain	*cis*-regulatory element involved in endosperm expression	GCN4_motif	CAAGCCA	+956, -55
	cis-acting regulatory element involved in seed-specific regulation	RY-element	CATGCATG	-27
	cis-acting regulatory element required for endosperm expression	Skn-1_motif	GTCAT	+209, -563
Hormone	*cis*-acting regulatory element involved in the MeJA-responsiveness	CGTCA-motif	CGTCA	+541, +552
	cis-acting regulatory element involved in the MeJA-responsiveness	TGACG-motif	TGACG	-541, -552
	cis-acting element involved in the abscisic acid	ABRE	TACGTG	-725
	gibberellin-responsive element	GARE-motif	TCTGTTG	-163
Environment/ Defense	*cis*-acting regulatory element essential for the anaerobic induction	ARE	TGGTTT	-479
	cis-acting element involved in defense and stress responsiveness	TC-rich	ATTTTCTTCA	-871
MYB binding	MYB-recognizing element	MRE	ANCNNCC	-230, +245, +311, -773, +849, +877, +920
bHLH binding	bHLH-recognizing element	BRE	CACN(A/C/T)(G/T)	+40, +393, -452, +462, +639, +702, +727, -944, +969

+, distance from ATG in a positive DNA strand; -, distance from ATG in a negative DNA strand.
